# Cell autonomous and non-autonomous functions of plant intracellular immune receptors in stomatal defense and apoplastic defense

**DOI:** 10.1371/journal.ppat.1008094

**Published:** 2019-10-25

**Authors:** Jiapei Yan, Huiyun Yu, Bo Li, Anqi Fan, Jeffrey Melkonian, Xiue Wang, Tong Zhou, Jian Hua

**Affiliations:** 1 School of Integrative Plant Science, Plant Biology Section, Cornell University, Ithaca, NY, United States of America; 2 Key Laboratory of Food Quality and Safety, Institute of Plant Protection, Jiangsu Academy of Agricultural Sciences, Nanjing, China; 3 School of Applied Physics and Engineering, Cornell University, Ithaca, NY, United States of America; 4 State Key Lab of Crop Genetics and Germplasm Enhancement, Nanjing Agricultural University, Nanjing, China; 5 School of Integrative Plant Science, Crop and Soil Sciences, Cornell University, Ithaca, NY, United States of America; Ohio State University, UNITED STATES

## Abstract

Stomatal closure defense and apoplastic defense are two major immunity mechanisms restricting the entry and propagation of microbe pathogens in plants. Surprisingly, activation of plant intracellular immune receptor NLR genes, while enhancing whole plant disease resistance, was sometimes linked to a defective stomatal defense in autoimmune mutants. Here we report the use of high temperature and genetic chimera to investigate the inter-dependence of stomatal and apoplastic defenses in autoimmunity. High temperature inhibits both stomatal and apoplastic defenses in the wild type, suppresses constitutive apoplastic defense responses and rescues the deficiency of stomatal closure response in autoimmune mutants. Chimeric plants have been generated to activate NLR only in guard cells or the non-guard cells. NLR activation in guard cells inhibits stomatal closure defense response in a cell autonomous manner likely through repressing ABA responses. At the same time, it leads to increased whole plant resistance accompanied by a slight increase in apoplastic defense. In addition, NLR activation in both guard and non-guard cells affects stomatal aperture and water potential. This study thus reveals that NLR activation has a differential effect on immunity in a cell type specific matter, which adds another layer of immune regulation with spatial information.

## Author summary

Plant immunity against foliar pathogens consists of stomatal defense to restrict the entry of pathogens and apoplastic defense to restrict the propagation of pathogens. Plant intracellular immune receptor NLR genes are known to trigger apoplastic defense upon perception of pathogen effectors. In this study, we uncovered a previously unidentified inhibitory role of NLR activation in stomatal defense by using autoimmune mutants where NLR genes are activated without pathogen trigger. We further showed by chimera plants that NLR function is cell autonomous in guard cells for stomatal closure defense and non-cell autonomous for apoplastic defense. In addition, we revealed that high temperature slows down stomatal closure defense in addition to its known effect of inhibiting apoplastic defense. This study therefore reveals an unexpected regulation of stomatal defense by NLR genes and provides the spatial information of these regulations in stomatal defense and apoplastic defense.

## Introduction

Plant immune responses have multiple phases targeting various stages of pathogen infections. Most microbial pathogens including bacteria, oomycete, and fungi gain access to intercellular spaces for growth through wounding sites or natural openings such as stomata [1]. Restriction of pathogen entry by stomatal closure upon perception of pathogen is the first line of immune responses and has been termed ‘stomatal defense’ [1–4]. This stomatal defense has been known as part of the PAMP (pathogen-associated molecular patterns)-triggered immunity (PTI). Recognition of PAMP by pattern recognition receptors induces calcium signaling and MAP kinase phosphorylation that affect the activities of channels to control stomatal closure [5–9]. Stomatal defense can be inhibited by pathogen-produced compounds that interfere with plant immunity proteins and signaling molecules. For instance, pathogens could produce a phytotoxin coronatine to induce stomatal re-opening through mimicking plant hormone jasmonic acid-isoleucine [4,10]. After gaining entry into the intercellular space of plants, pathogens encounter another plant defense mechanism, apoplastic defense, that restricts their propagation [11,12]. This apoplastic defense delivers reactive oxygen species, toxic compounds, and anti-pathogen protein molecules such as ‘Pathogenesis Related’ (PR) proteins to the apoplastic space. The upregulation of genes coding for these molecules and their secretary pathway is part of the transcriptional reprogramming in both PTI and another layer of immune responses named ‘effector-triggered immunity’ (ETI) [7]. ETI recognizes pathogen secreted effectors (that are intended to inhibit PTI) with plant intracellular immune receptor proteins, or resistance (R) proteins, and has a much stronger and faster immune response than that in PTI [13]. Most of these immune receptors are nucleotide-binding leucine-rich repeat proteins (NLR), some of which are indirectly involved in transcriptional control. Activation of NLR genes leads to a strong apoplastic defense and even local programmed cell death to restrict the spread of pathogens.

Stomatal defense has been mostly studied under the context of PTI for stomatal closure responses [14]. A timely closure of stomata is considered to be the first line of defense against pathogen infection. A number of genes have been shown to be required for stomatal closure in response to pathogens and thus positively contribute to overall plant disease resistance as measured by reduced pathogen propagation in apoplastic space. For example, loss-of-function (LOF) mutants of PAMP receptor FLS2 or signaling components MAPK3/6 are defective in MAMP triggered stomatal closure and have reduced resistance to *Pseudomonas syringae* [15]. Recent genetic screens using a pathogen strain deficient in coronatine production and thus disarmed of combating stomatal defense revealed positive regulators affecting only stomatal defense or only apoplastic defense [16]. Surprisingly, some immune regulators are found to have opposite effects on stomatal closure response and whole plant disease resistance. For example, mutants defective in ABA biosynthesis are compromised in stomatal defense [4] but could gain enhanced whole plant disease resistance [17]. This opposing function of ABA is reconciled by temporal separation in distinct pre-invasion and post-invasion phases of pathogen infection [18]. One other potential example is the NLR gene *SNC1* whose constitutive active form *SNC1-1* induces enhanced apoplastic defense [19] but inhibits stomatal closure response to ABA although the stomatal defense in response to pathogen has not been tested [20]. The more established examples are calcium pumps ACA10 and ACA8 as well as their interacting calcium binding protein BON1, all of which possess contrasting roles in stomatal defense and whole plant disease resistance. The LOF mutants of *ACA10* and *ACA8* are defective in stomatal closure response to pathogens but exhibit enhanced resistance to the virulent bacterial pathogen *Pseudomonas syringae pv*. *tomato* (*Pst*) DC3000 compared to the wild type [21]. Similarly, the LOF mutants of *BON1* do not close stomata in response to pathogens but are more resistant to *Pst* DC3000 than the wild type [22,23]. The mutants of *bon1-1*, *aca10/8*, and *snc1-1* are named autoimmune mutants because defense responses for restricting pathogen growth are turned on in the absence of pathogen infection. This upregulation of defense responses in *bon1* or *aca10/8* mutants is conferred by or associated with upregulation of *NLR* gene activities. The Col-0 accession specific *NLR* gene *SNC1* is upregulated in the *bon1-1* mutant under normal growth condition and confers enhanced disease resistance. A No-0 specific *CIF2* gene that confers a constitutive defense response in the *aca10-1* mutant is also likely to be an *NLR* gene based on its *NLR*-associated features such as accession-specificity, *PAD4-*dependence and temperature-dependence [21]. Therefore, *ACA10* and *BON1* are positive regulators of stomatal defense but negative regulators of NLR and whole plant apoplastic disease resistance. The opposite roles of NLR regulators *BON1* and *ACA10* in stomatal defense and apoplastic defense is intriguing. Does NLR gene activation have opposing roles in stomatal defense and apoplastic defense? Are these two roles executed independently in separate space and/or phases of defense responses? If one defense influences the other, how is the communication achieved especially when they are spatially separated?

Environmental factors such as light and temperature have large impacts on plant-pathogen interactions, adding a layer of environmental regulation on top of the layer of genetic determinants from plants and pathogens. Temperature affects both pathogen virulence and plant immunity [24–26], and it is an important determinant for disease epidemics [27,28]. The interplay between temperature and plant immunity is multi-layered and is dependent on the type of immunity involved [29]. ETI signaling is often inhibited by a high temperature [30], while PTI signaling is reported to be activated by high temperature [24]. The suppression of resistance mediated by NLRs such as SNC1 and RPS4 by high temperature 28°C (versus normal growth temperature 22°C) is associated with a reduction of nuclear accumulation and thus activities of these NLR proteins [17,31]. The suppression of immunity by high temperature is also associated with a reduction of the pathway of salicylic acid (SA). Expression of genes indicative of SA signaling is lower at high temperature [31,32]. Elevated temperature inhibits pathogen-induced SA biosynthesis, and application of the SA analogue Benzothiadiazole (BTH) can enhance resistance at high temperature, indicating that pathogen-induced SA production is a temperature-sensitive step in the SA defense network [25]. In contrast to SA, the production of abscisic acid (ABA) is not significantly reduced at high temperature. However, ABA has a negative regulation on *NLR* gene-mediated resistance, and ABA deficiency enhances disease resistance at high temperature [17].

Here we investigated the inter-dependence between stomatal and apoplastic defenses and cell autonomy of each defense. Specifically, stomatal and apoplastic defenses were examined in mutants related to *SNC1* and *BON1* to determine the correlation of these two defenses by two means. One was through different temperature conditions and the other was through chimera plants to assess cell autonomy of these molecules and the communication between guard cells and the rest of the plants. Results from these studies indicate that NLR activation in guard cells inhibits stomatal defense in a cell-autonomous manner, which is associated with repression of ABA response. In addition, NLR activation in guard cells and non-guard cells could have non-cell autonomous effects to impact whole plant defense and stomatal behavior respectively.

## Materials and methods

### Plant materials and growth condition

The *Arabidopsis* plants were grown under a 12 hour (hr) white light condition with photon density at 100 μmol m^−2^ s^−1^ and relative humidity at 50% to 70% for stomatal aperture assays, pathogen growth assays, leaf water potential measurements and gene expression studies unless stated otherwise. Plants were grown at either 22°C or 28°C for the entire time, except in the stomatal closure response assay where plants were grown first at 22°C followed by three days of 28°C growth before the 28°C assay.

### Plasmid construction

For functional complementation in guard cells, sequences of 1702 bps upstream of the start codon of *GC1* (At1g22690) [33] was amplified from genomic DNA using oligos listed in S1 Table. The PCR amplified fragment of the *GC1* promoter (*pGC1*) was digested by KpnI and AscI and then ligated into the Gateway (GW) pMDC99 vector [34]. The resulted pMDC99-pGC1 was used to generate constructs for expressing *BON1* and *SNC1-1* in guard cells. The full-length cDNA of *BON1* was amplified using oligos listed in Supplemental S1 Table and inserted into the GW entry vector pCR8 TOPO TA vector (Invitrogen). The *BON1* gene was transferred from the entry clone to pMDC99-pGC1 and transformed into the *Agrobacterium tumefaciens* strain GV3101 for plant transformation. The full-length *SNC1-1* was amplified from the genomic DNA of the *snc1-1* mutant using oligos listed in S1 Table and constructed into pMDC99-pGC1 by the Gateway cloning method described above.

### Stomatal aperture assay

Stomatal closure assay was performed as previously described [2] with minor modifications. The 5^th^ to 7^th^ rosette leaves from 4-week-old *Arabidopsis* plants were detached and floated on the stomatal opening buffer for 1.5 hr under the same growth condition. Leaf peels were then collected and incubated in the stomatal opening buffer or buffer with either ABA (20 μM), SA (20 μM), *Pst* DC3000 or *Pst* DC3000 COR^-^ (OD_600_ = 0.2) on slides in petri dishes with lid on and incubated in the same growth condition. For the steady-state stomatal assays, leaf peels were collected and incubated in H_2_O. The leaf peels were then observed under a light microscope. Images were taken and at least 30 stomata were recorded for each sample. Stomatal apertures were measured with ImageJ and calculated as the ratio of the inner width/outer length of each pair of guard cells.

### Pathogen growth assay

*Pst* DC3000 cells grown on plates with King’s B medium were collected and diluted with 10 mM MgCl_2_ (for syringe infiltration) or with 10 mM MgCl_2_ and 0.02% Silwet L-77 (for dipping inoculation). Syringe infiltration was performed as previously described [35]. Bacteria were diluted to an OD_600_ of 0.0002 and syringe-infiltrated into the 5^th^ and 6^th^ leaves of 4-week-old plants. Dipping inoculation was performed as previously described [23]. Bacteria were diluted to OD_600_ of 0.05 and dip-inoculated into two-week-old seedlings.

### Quantitative real-time PCR (qPCR) and expression data analysis

Leaf tissues were collected from 5-week-old plants and were flash-frozen in liquid nitrogen before homogenization using a pestle. Total RNA extraction and the cDNA synthesis were performed as previously described [36]. The qPCR was carried out using the CFX96 real-time PCR system (Bio-Rad), primer sequences used for gene amplification were listed in S1 Table. Relative expression of each gene was normalized to the expression of *ACTIN 2* in the same cDNA sample. The fold difference (2^-ΔΔCt^) was calculated using the CFX Manager Software, version 1.5 (Bio-Rad).

### Enrichment of guard cells and isolation of mesophyll cells

Leaves were collected from 5-week-old plants grown under 12 hr/12 hr L/D condition and the central veins were removed by razor blade. Approximately 80 leaves from 18 plants were pooled for each genotype for guard cell enrichment according to the ice-blender method described previously [37].

For isolating mesophyll cells, fully expanded leaves were detached from 5 to 6 weeks old plants that were grown under 12 hr/12 hr L/D condition. Mesophyll cells were harvested according to the protocol previously described [38].

### Leaf water potential (LWP) measurement

Leaves were collected at the end of the dark cycle from plants of 4 to 5 weeks old and LWP was measured with the pressure chamber as described by Campbell (1985) [39]. Leaf water potentials are reported in megapascals (MPa) where 1 MPa = 10 bars, and are reported as negative values, following the convention used in plant water relations research.

### Statistical analysis

Data of stomatal aperture, pathogen growth, gene expression and leaf water potential were subjected to a one-way ANOVA followed by student’s t test, Tukey-Kramer test or Duncan’s new multiple range test as indicated to assess differences between samples. Significance was defined by *p* value as stated.

## Results

### Temperature alters stomatal response to pathogen in *A*. *thaliana*

**Fig 1:**
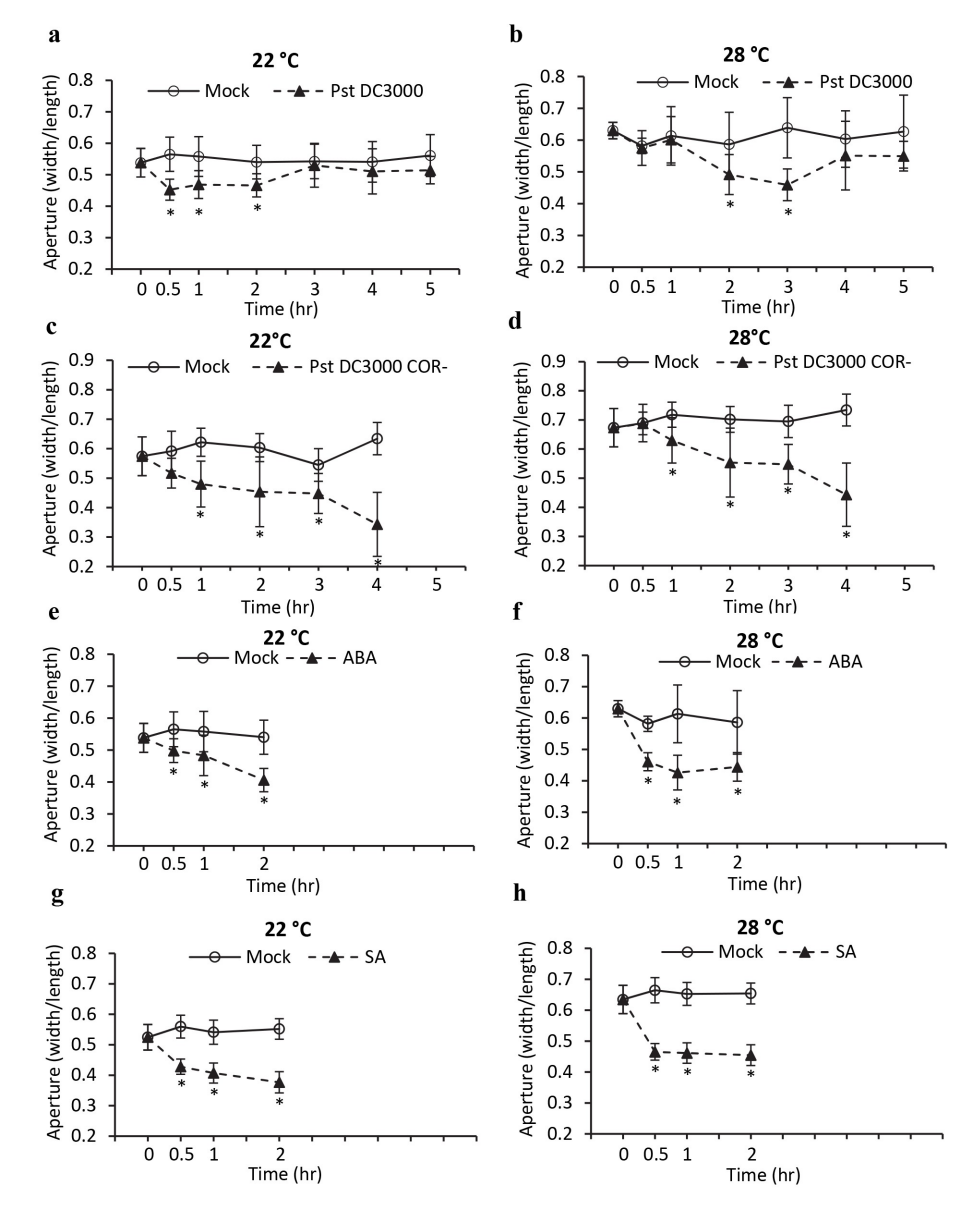
Temperature affects stomatal movements in *A*. *thaliana*. Shown are stomatal apertures (defined by the ratio between width and length) across sequential time points at 22°C and 28°C. The leaf abaxial epidermal layer was incubated in the opening buffer (Mock) or buffer with *Pseudomonas syringae pv*. *tomato* (*Pst*) DC3000 (**a**, **b**), *Pst* DC3000 COR-deficient (**c**, **d**), 20 μM ABA (**e**, **f**) or 20 μM SA (**g**, **h**). Results represent two biological repeats. Error bars indicate standard deviations (SDs) (n = 30 stomata). Asterisks indicate statistically significant differences between mock and treatment (*p*<0.001, student’s t test).

To investigate the connection of stomatal defense and apoplastic defense, we first determined whether or not environmental factors that affect apoplastic defense could also affect stomatal defense responses. Specifically, we tested the effect of high temperature on stomatal movement in response to pathogen as high temperature has been shown to inhibit NLR mediated apoplastic defense but enhances PTI. To this end, stomata in the abaxial epidermal layer were chemically opened and incubated with virulent bacterial pathogen *Pst* DC3000 at 22°C and 28°C. Stomatal aperture was then measured at 0.5, 1, 2, 3, 4, and 5 hr after incubation. At 22°C, stomata closed at 0.5 hr and re-opened after 3 hr when incubated with *Pst* DC3000 (Fig 1A). This data is consistent with the previous report on the stomatal closure induced by PTI and re-opened by virulent factor coronatine [4]. At 28°C, stomata did not close at 0.5 or 1 hr when they would do at 22°C but closed at a later time point of 2 hr upon *Pst* DC3000 treatment (Fig 1B). They reopened at 4 hr at 28°C, also later than they would at 22°C after *Pst* DC3000 treatment (Fig 1A and 1B). Coronatine deficient strain *Pst* DC3000 COR^-^ (which does not cause stomatal reopening) was further used to assess stomatal movement under different temperatures. As expected, stomata closed at 0.5 hr and were kept closed at 4 hr at 22°C after treatment with COR^-^ strain. At 28°C, stomata did not close till 1 hr after treatment and were still closed at 4 hr (Fig 1C and 1D).

We then determined if this delay in closure at higher temperature is specific to pathogens or is a general feature of stomatal response by assaying ABA- and SA-induced stomatal closure at 22°C and 28°C. Stomata exposed to 20 μM of ABA closed significantly after 0.5 hr at both temperatures, with minimum aperture occurring earlier at 28°C (1 hr) compared to 22°C (2 hr) (Fig 1E and 1F). SA is known to cause stomatal closure at normal temperatures [2,40]. Indeed, stomata closed in response to 20 μM of SA at 0.5 hr at 22°C and reached its maximum closure at 2 hr (Fig 1G). At 28°C, stomata closed at 0.5 hr, already reaching its maximum (Fig 1H). Therefore, high temperature delays stomatal closure response to pathogens but slightly quickens the closure response to ABA and SA.

### Elevated temperature suppresses stomatal closure defect in the autoimmune mutants

We used four autoimmune mutants to test the correlation between stomatal defense and apoplastic defense. Three of them, *bon1-1*, *snc1-1* and *aca10 aca8* have higher apoplastic defense due to activated *NLR* genes. The fourth one *aca4 aca11* is a double LOF mutant of the ER localized calcium pumps ACA4 and ACA11. It exhibits autoimmune responses [36,41] but it is not determined if its enhanced apoplastic defense is NLR related.

Apoplastic resistance was measured for these four autoimmune mutants at 22°C and 28°C. Consistent with the previous findings, *bon1-1* and *snc1-1* had highly reduced while *aca10 aca8* and *aca4 aca11* had moderately reduced growth of the virulent pathogen *Pst* DC3000 compared to the wild-type Col-0 at 22°C (S1A and S1C Fig). As previously reported, the elevated resistance in *bon1-1*, *snc1-1*, and *aca10 aca8* was suppressed partially or totally at 28°C compared to 22°C (S1B and S1D Fig). A suppression by 28°C was similarly observed for *aca4 aca11* (S1C and S1D Fig). Therefore, elevated temperature suppresses the enhanced apoplastic disease resistance in all these autoimmune mutants (S2 Table). This is consistent with the reports that activities or expression of NLR genes are higher in *snc1-1*, *bon1-1*, *aca10 aca8* at 22°C and NLR activities could be repressed at 28°C. It also suggests that autoimmunity of *aca4 aca11* could be associated with NLR activation.

**Fig 2:**
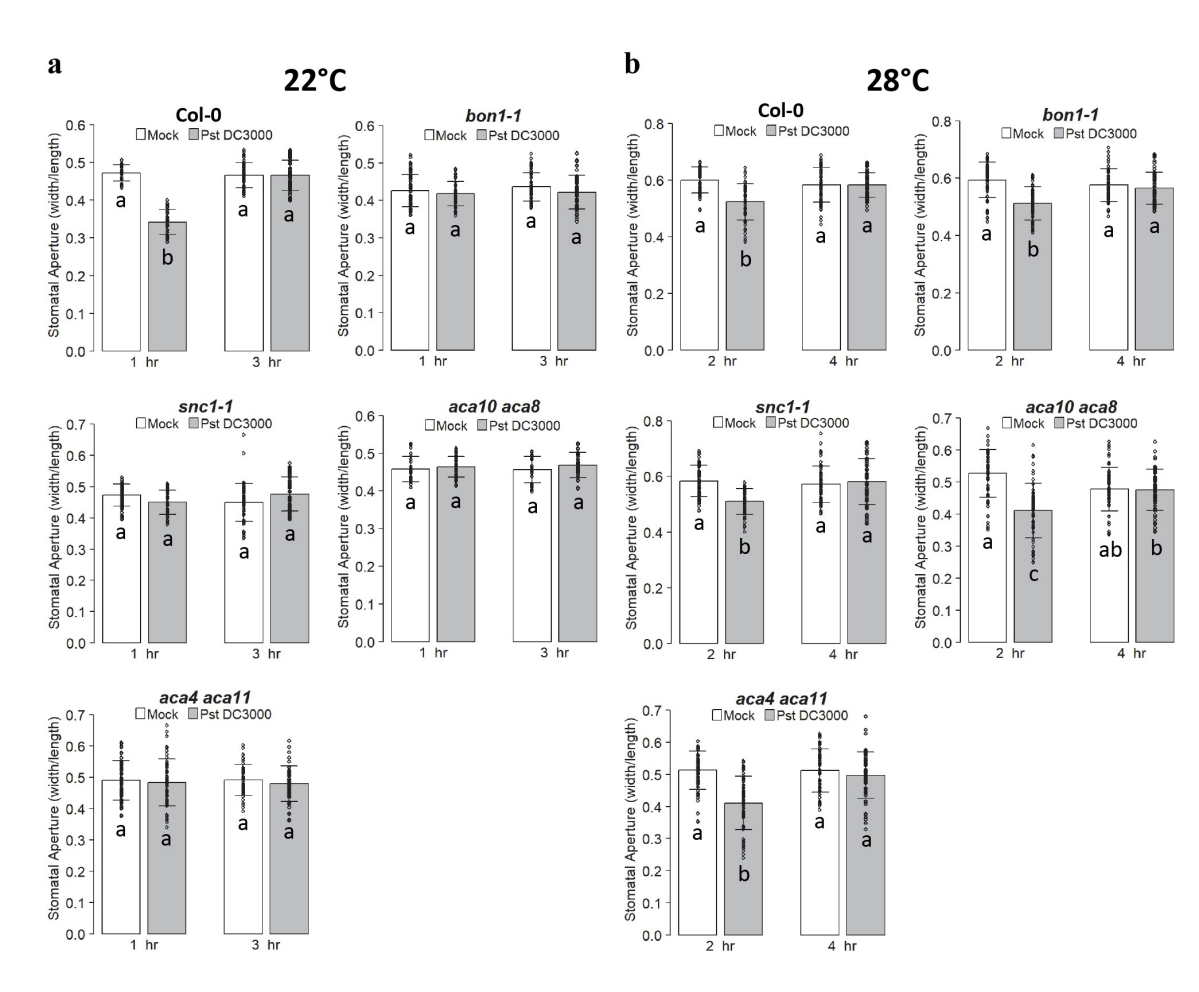
Mutants *bon1-1*, *snc1-1*, *aca10 aca8* and *aca4 aca11* exhibit a temperature-dependent stomatal closure defect in response to *Pst* DC3000. Shown are stomatal apertures in response to *Pst* DC3000 or buffer alone (mock) in the autoimmune mutants *bon1-1*, *snc1-1*, *aca10 aca8* and *aca4 aca11* as well as the wild-type Col-0 at 22°C (**a**) and 28°C (**b**). Biological duplicates were averaged and statistically analyzed with one-way Anova followed by Tukey-Kramer test. Different letters indicate statistically significant difference (*p*< 0.001) and error bars indicate SDs (n = 60 stomata).

We then analyzed stomatal defense of *bon1-1*, *snc1-1*, *aca10 aca8* and *aca4 aca11* at 22°C and 28°C. After treatment with *Pst* DC3000, stomatal aperture was analyzed at 1 hr and 3 hr for the 22°C samples and 2 hr and 4 hr for the 28°C samples because the wild type Col-0 had significant closure and re-opening responses at these two time points respectively (Fig 1A and 1B). In contrast to the wild type, *bon1-1* and *aca10/8* did not close their stomata in response to *Pst* DC3000 at 1 hr and remained open at 3 hr, consistent with previous reports. Similarly, stomatal closure was not observed in the mutants of *snc1-1* or *aca4 aca11* (Fig 2A). Interestingly, elevated temperature restored a wild-type stomatal response in all four mutants: closed stomata at 2 hr and reopened stomata at 4 hr (Fig 2B). This indicates that a high temperature suppressed the stomatal defense defects (no closure response) as well as the apoplastic defense defects (constitutive active defense) in these mutants (S2 Table).

**Fig 3:**
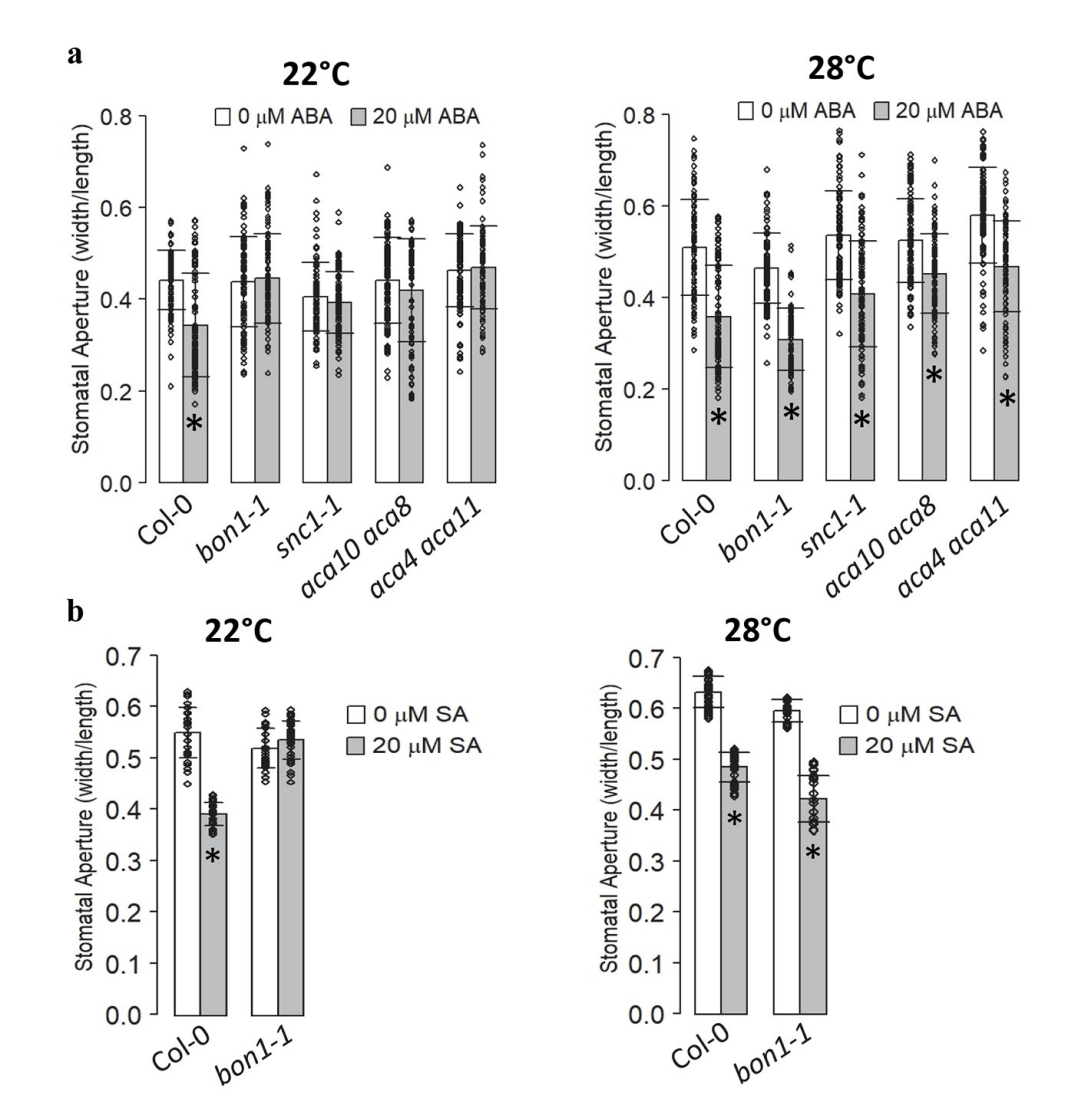
Autoimmune mutants exhibit a temperature-dependent stomatal closure defect in response to ABA and SA. Shown are stomatal apertures in response to 20 μM ABA (**a**) or SA (**b**) at 22°C and 28°C after 1.5 hr incubation. Biological triplicates (**a**, n = 90 stomata) or duplicates (**b**, n = 60 stomata) were averaged and statistically analyzed with Student’s t test. Asterisks indicate statistically significant differences in stomata aperture between control and treatment (*p*< 0.001). Error bars indicate SDs.

To determine if the stomatal response defect in these mutants are specific to pathogens, we examined their response to ABA in *bon1-1*, *snc1-1*, *aca10 aca8* and *aca4 aca11* mutants. Stomatal apertures were measured at 1.5 hr after ABA treatment. None of these mutants were responsive to ABA at 22°C, but they were all responsive to ABA at 28°C (Fig 3A and S2 Table). SA response was also assayed in stomata for the *bon1-1* mutant. Unlike the wild type, the *bon1-1* mutant did not close stomata at 1.5 hr after SA treatment at 22°C. However, it closed stomata at 1.5 hr in response to SA similarly to the wild type at 28°C (Fig 3B). Together, these results indicate that *BON1*, *SNC1*, *ACA8*, *ACA10*, *ACA4* and *ACA11* all have opposite effects on stomatal closure defense and apoplastic defense at 22°C, but they do not have a significant impact on either defense at elevated temperature. Therefore, the stomatal defense deficiency is tightly associated with apoplastic defense enhancement in these autoimmune mutants.

### The *SNC1* active allele has a cell autonomous activity in guard cells

**Fig 4:**
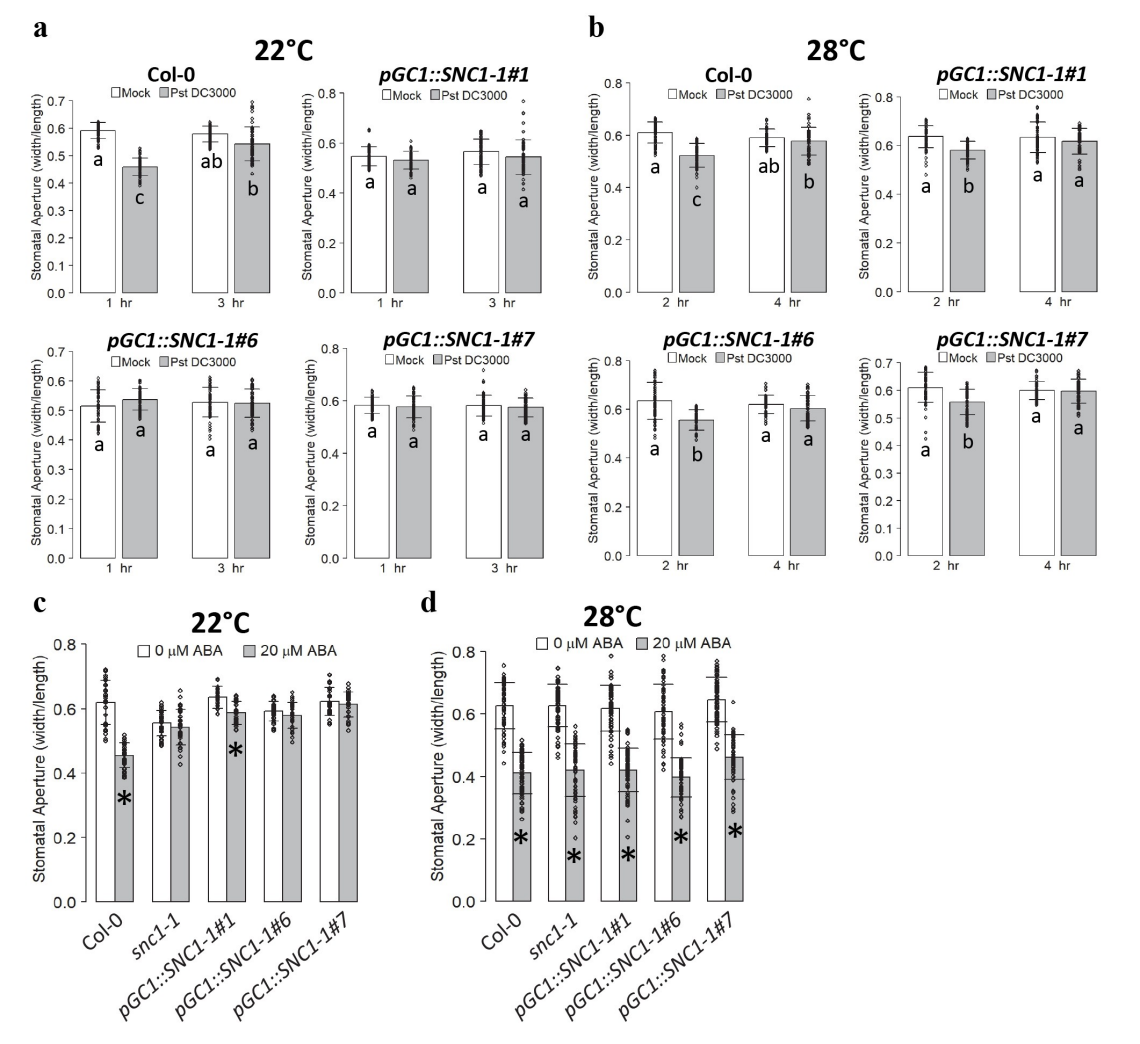
Expressing the active mutant gene *SNC1-1* in guard cells results in stomatal closure defect. (**a**, **b**) Shown are stomatal apertures in response to *Pst* DC3000 or buffer alone (mock) in Col-0 and three independent *pGC1*::*SNC1-1* transgenic lines (T3 generation) at 22°C (**a**) and 28°C (**b**) at indicated time points. Biological duplicates were averaged and statistically analyzed with one-way Anova followed by Tukey-Kramer test. Different letters indicate statistically significant difference (*p*< 0.001), error bars indicate SDs (n = 60 stomata). (**c**, **d**) Shown are stomatal apertures in response to 20 μM ABA or buffer alone (0 μM ABA) in Col-0, *snc1-1* and the three *pGC1*::*SNC1-1* lines at 22°C (**c**) and 28°C (**d**) after 1.5 hr. Biological duplicates were averaged and statistically analyzed with Student’s t test. Asterisks indicate statistically significant differences in stomata aperture between 0 and 20 μM ABA (*p*< 0.001). Error bars indicate SDs (n = 60 stomata).

We tested whether or not the stomatal closure defense and apoplastic defense could be separated by creating two types of chimeric plants where the guard cells and the rest part of the plants have different genotypes of *SNC1* or *BON1*. The first chimera we made is an otherwise wild-type plant except for being *snc1-1* in guard cells. This was achieved by expressing the active mutant form of *SNC1*, *SNC1-1*, by the guard-cell specific *pGC1* promoter in the wild-type Col-0. The specificity of the *GC1* promoter to drive guard cell expression was reported earlier [33] and tested in the second chimera as detailed later. We first analyzed stomatal responses to pathogens and ABA in three independent *pGC1*::*SNC1-1* transgenic lines. At 1 hr after incubation with *Pst* DC3000 at 22°C, all three lines displayed no closure at all compared to the wild type exhibiting 25% stomatal closure, while at 3 hr after incubation, stomata of *pGC1*::*SNC1-1* lines remained open and stomata of Col-0 re-opened (Fig 4A). As expected, all *pGC1*::*SNC1-1* lines had a wild-type response to *Pst* DC3000 at 28°C, closing and re-opening their stomata at 2 hr and 4 hr, respectively (Fig 4B). In response to ABA, these lines all exhibited a defect in stomatal closure at 22°C. Lines ^#^6 and ^#^7 did not show closure at 1 hr and line ^#^1 had a much reduced closure (8% of Mock) compared to the wild-type Col-0 (27% of Mock) (Fig 4C). As expected, the *pGC1*::*SNC1-1* lines had wild-type response to ABA at 28°C (Fig 4D). Therefore, the expression of the active form of *SNC1* gene in guard cells is sufficient to cause stomatal closure defect in response to pathogen and ABA, and this defect is temperature-dependent (S2 Table).

### Influence on whole plant disease resistance from stomatal activity of *SNC1*

**Fig 5:**
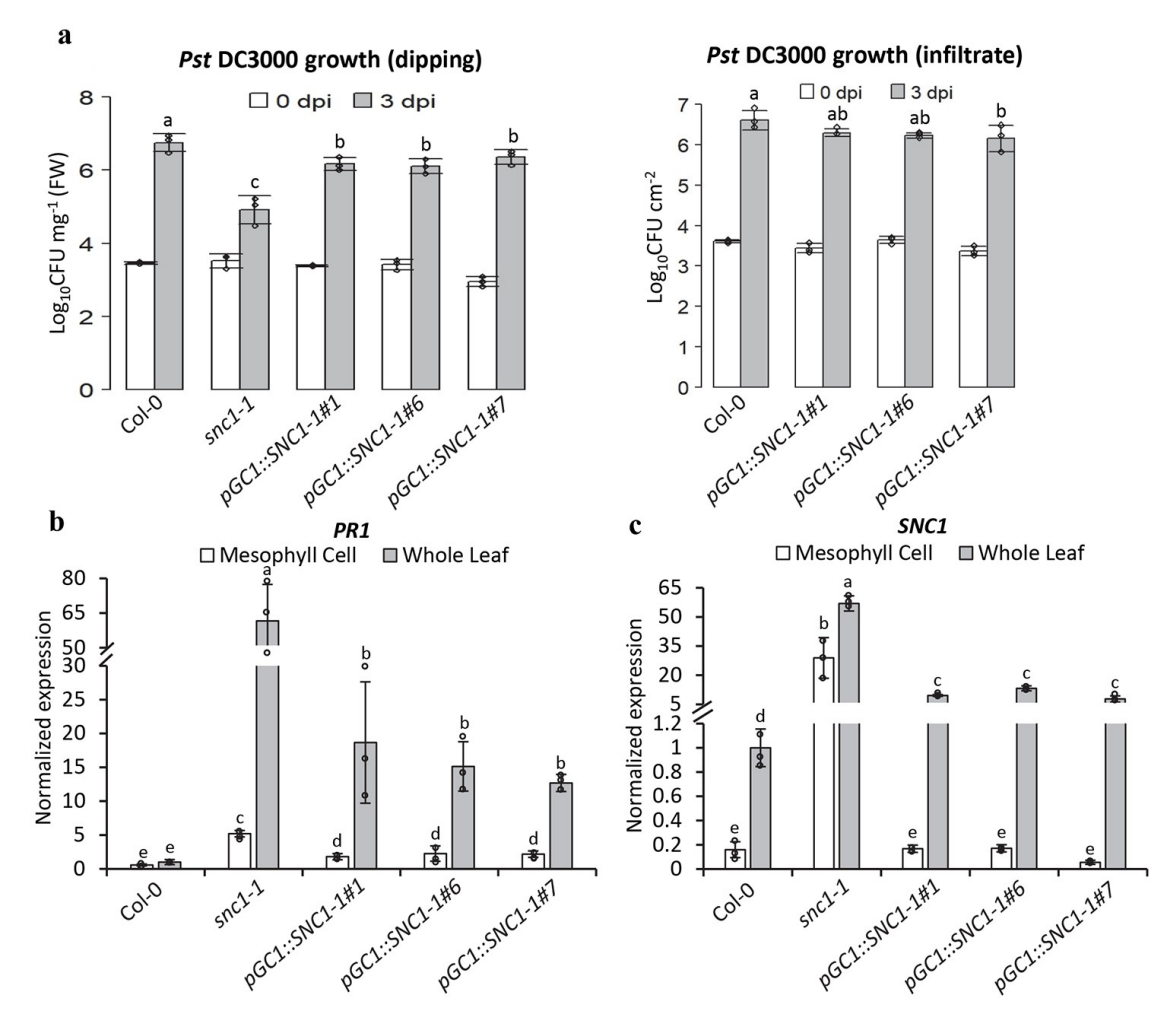
Expressing the active mutant gene *SNC1-1* in guard cells enhances whole plant resistance to *Pst* DC3000. (**a**) Growth of *Pst* DC3000 at 0 dpi and 3 dpi after dipping or infiltration inoculation. Results are from three biological repeats in one experiment representative of two independent experiments. Error bars indicate SDs. Different letters indicate statistically significant differences in pathogen growth between different genotypes (*p*< 0.05, student’s t test). (**b**, **c**) Transcript abundance of *PR1* (**b**) and *SNC1* (**c**) in mesophyll cells and whole leaves of indicated plant lines assayed by qPCR. Plants were grown for 5 weeks and RNA was harvested from mesophyll cells and rosette leaves. Results represent three biological replicates. The expression of target genes was normalized to reference gene *ACTIN*, and relative to their expression in whole leaves of Col-0 which was set as 1. Values are arithmetic means ± S.E. Different letters indicate statistically significant differences of the gene expression between indicated plant lines (*p*< 0.05, based on one-way ANOVA followed by Duncan’s new multiple range test).

We next investigated whether or not the expression of the active *SNC1-1* in guard cells alters the defense responses of the whole plant. Three *pGC1*::*SNC1-1* transgenic lines were inoculated with *Pst* DC3000 by dipping, and disease resistance in these lines was compared to those in Col-0 and *snc1-1*. At 3 dpi, *Pst* DC3000 amplified to 10^6.7^ CFU (colony forming unit) mg^-1^ FW (fresh weight) in the wild-type Col-0 but reached to only about 10^4.9^ in *snc1-1* (Fig 5A). It amplified to 10^6.2^, 10^6.1^, and 10^6.3^ CFU/mg^-1^ FW in lines ^#^1, ^#^6, ^#^7 of *pGC1*::*SNC1-1*, respectively (Fig 5A). Therefore, all three lines had a lower pathogen growth compared to Col-0, but a higher growth than *snc1-1* when inoculated by dipping (S2 Table). We also assayed pathogen growth by infiltration inoculation. In four independent experiments, significant increase of resistance was observed for *pGC1*::*SNC1-1* lines in two experiments but not the other two experiments. In one experiment with three biological repeats, a significant increase of disease resistance was observed in line #7 and a slight but not statistically significant increase was observed in lines #1 and #6 of *pGC1*::*SNC1-1* transgenic plants compared to the wild type (Fig 5A). Because the *pGC1*::*SNC1-1* lines are deficient in stomatal closure defense, these data indicate that they have increased whole plant resistance with a slight increase of apoplastic disease resistance.

Expression of defense related genes was then analyzed in *pGC1*::*SNC1-1* transgenic plants. RNAs were isolated from above-ground plants at 5 weeks old, and qPCR was used to measure gene expression. Consistent with the pathogen growth phenotype, defense response genes were upregulated in the transgenic plants. Quantitative RT-PCR analysis of the leaf tissues revealed that defense response gene *PR1* (*Pathogenesis Related Protein 1*) was significantly increased in all three *pGC1*::*SNC1-1* lines compared to the wild type Col-0 although it was lower than *snc1-1*. An increase of *SNC1* expression was also observed in leaves of all three *pGC1*::*SNC1-1* transgenic lines (Fig 5B and 5C). We further examined the expression of *PR1* and *SNC1* in mesophyll cells in the *pGC1*::*SNC1-1* lines. In the mesophyll cell preparations, a high expression of mesophyll cell specific *CAB3* gene and a low expression of epidermal specific *CER6* gene was found (S3A Fig), indicating little contamination of epidermal cells in the preparation. Interestingly, mesophyll *SNC1* expression in these lines was similar to that in wild type Col-0, but mesophyll *PR1* expression in all these lines was higher than in the wild type although lower than in *snc1-1*(Fig 5B and 5C). These results revealed that the mutant gene *SNC1-1* in guard cells results in stomatal defense deficiency, but at the same time slightly enhances apoplastic disease resistance of the whole plant accompanied by an upregulation of the mesophyll *PR1* gene expression (S2 Table). Although the expression of mesophyll and epidermal specific genes indicates a good purity of mesophyll cell preparation, it cannot be entirely excluded that a few mesophyll cells might express the mutant form of *SNC1-1* gene and induces cell autonomous instead of non-cell autonomous *PR1* expression. In any case, the enhanced resistance in the *pGC1*::*SNC1-1* was much milder than *snc1-1*, and no gross growth defect was observed in the *pGC1*::*SNC1-1* transgenic plants (S2 Fig).

### *BON1* has a cell autonomous activity in guard cells

The second and complementary chimeric plant was a *bon1-1* mutant except for the guard cells. This was achieved through expressing the wild-type *BON1* gene under a guard-cell specific promoter *pGC1* in the *bon1-1* mutant so that only guard cells have a functional *BON1*. To determine if *BON1* was indeed specifically expressed in guard cells in the chimera plants, we isolated RNAs from mesophyll cells of *pGC1*::*BON1/bon1* plants. Real time RT-PCR revealed that *GC1* was expressed in the whole leaf tissue but not in the mesophyll cells while *CAB3* was highly expressed and *CER6* was lowly expressed in the mesophyll cell preparations (S3B Fig). This indicates a good separation of epidermal cells from mesophyll cells. The expression of *BON1* could be detected from the whole leaves and mesophyll cells in the wild-type Col-0 but not in the *bon1-1* (S3B Fig). In all three *pGC1*::*BON1/bon1* transgenic lines, but not *bon1-1*, *BON1* expression was detected in whole leaves but not from mesophyll cells (S3B Fig). This data indicates that the expression of *BON1* driven by *pGC1* was indeed specific to guard cells (at the resolution of epidermal cells) in these transgenic lines.

**Fig 6:**
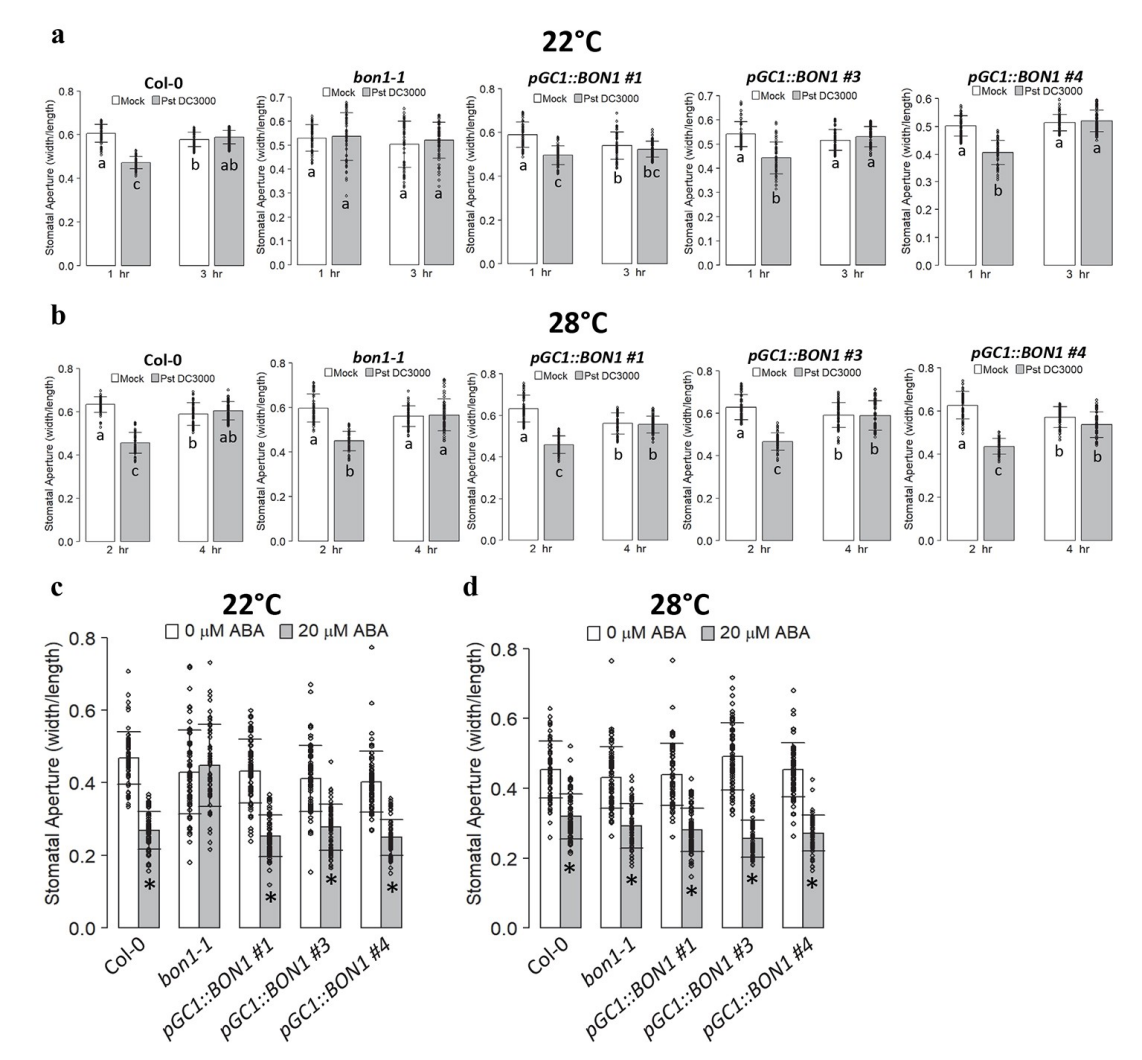
Expressing *BON1* in guard cells restores the wild-type stomatal responses in *bon1-1*. (**a**, **b**) Shown are stomatal apertures in response to *Pst* DC3000 or buffer alone (mock) in Col-0, *bon1-1* and three independent *pGC1*::*BON1/bon1* transgenic lines (T3 generation) at 22°C (**a**) and 28°C (**b**) at indicated time points. Biological duplicates were averaged and statistically analyzed with one-way Anova followed by Tukey-Kramer test. Different letters indicate statistically significant difference (*p*< 0.001), error bars indicate SDs (n = 60 stomata). (**c**, **d**) Shown are stomatal apertures in response to 20 μM ABA or buffer alone (0 μM ABA) in Col-0, *bon1-1* and three independent *pGC1*::*BON1/bon1* transgenic lines (T2 generation) at 22°C (**c**) and 28°C (**d**) after 1.5 hr. Biological triplicates were averaged and statistically analyzed with Student’s t test. Asterisks indicate statistically significant differences in stomata aperture between 0 and 20 μM ABA (*p*< 0.001). Error bars indicate SDs (n = 90 stomata).

We then analyzed the stomatal response in these chimeric *pGC1*::*BON1/bon1* transgenic plants. Homozygous plants of three independent transgenic lines were measured for their stomatal response to the virulent pathogen *Pst* DC3000. As expected, we observed stomatal closure in Col-0 after 1 hr and re-opening after 3 hr incubation with *Pst* DC3000 while no closure was observed in *bon1-1* in response to *Pst* DC3000 at 22°C (Fig 6A). The *pGC1*::*BON1/bon1* transgenic plants behaved like the wild type: closure at 1 hr and re-opening at 3 hr after incubation with *Pst* DC3000 (Fig 6A). At 28°C, all *pGC1*::*BON1/bon1* lines were sensitive to *Pst* DC3000, closing and re-opening at 2 hr and 4 hr, respectively (Fig 6B). Responses to ABA were also measured in these chimeric plants. The *pGC1*::*BON1/bon1* transgenic plants closed their stomata in response to ABA similarly to Col-0; whereas *bon1-1* plants did not close theirs in response to ABA at 22°C. (Fig 6C). As expected, all plant lines were responsive to ABA at 28°C (Fig 6D). Therefore, the expression of *BON1* in guard cells is sufficient to restore the wild-type stomatal response to pathogen and ABA in *bon1-1* (S2 Table), and the stomatal defense function of *BON1* is cell autonomous.

### Influence on whole plant disease resistance from stomatal activity of *BON1*

**Fig 7:**
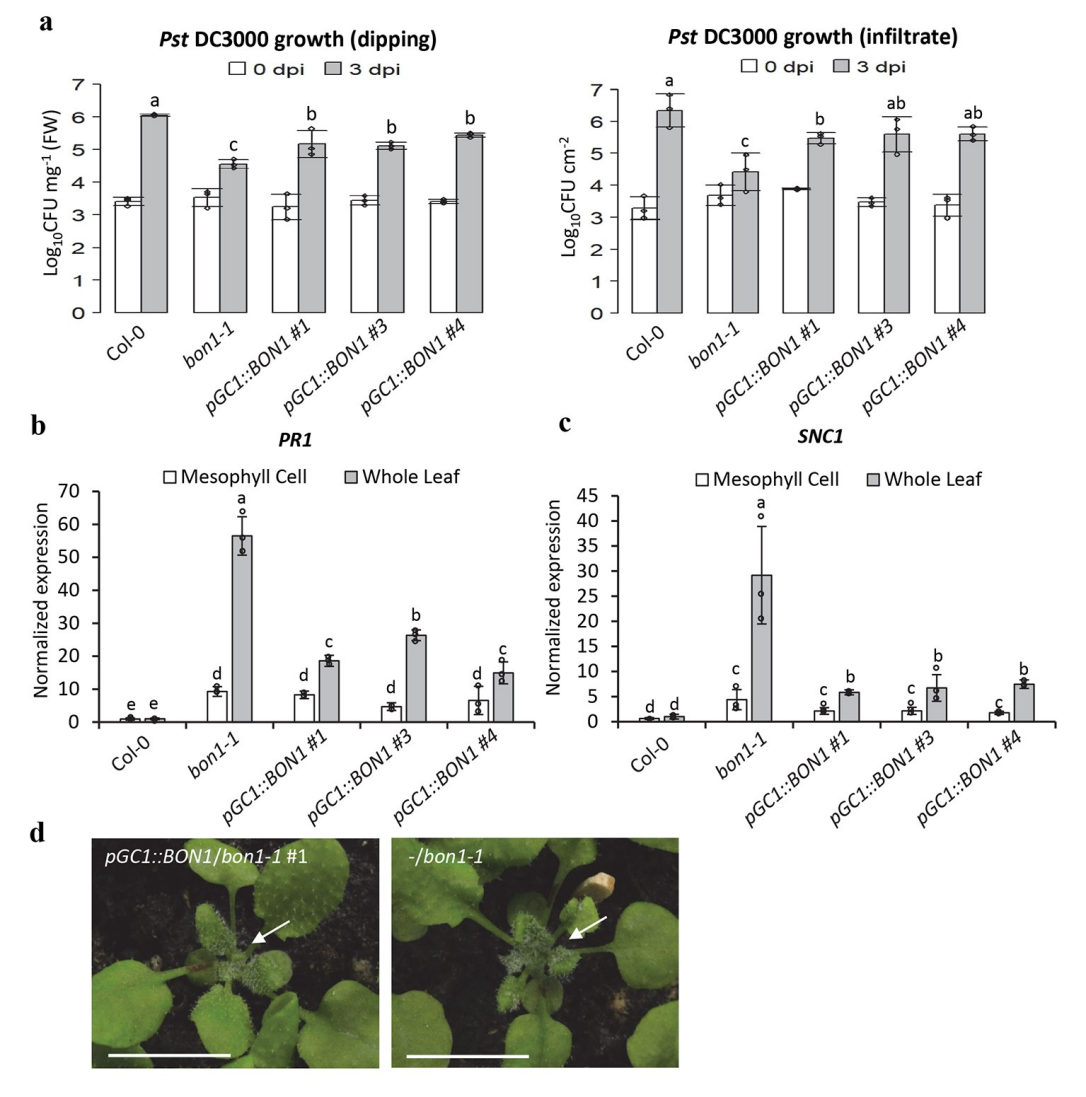
Expressing *BON1* in guard cells reduces autoimmunity in *bon1-1*. (**a**) Growth of *Pst* DC3000 at 0 dpi and 3 dpi after dipping or syringe-infiltration. Results are from three biological repeats in one experiment representative of two independent experiments. Error bars indicate SDs (n = 3). Different letters indicate statistically significant differences in pathogen growth between different genotypes (*p*< 0.05, student’s t test). (**b**, **c**) Transcript abundance of *PR1* (**b**) and *SNC1* (**c**) in mesophyll cells and whole leaves assayed by quantitative Real-time PCR (qPCR). Plants were grown for 5 weeks at 22°C and RNA was harvested from mesophyll cells or rosette leaves, respectively. Results represent three biological replicates. The expression of target genes was normalized to reference gene *ACTIN*, and relative to their expression in whole leaves of Col-0 which was set as 1. Values are arithmetic means ± standard error (S.E.). Different letters indicate statistically significant differences between indicated plant lines (*p*< 0.05, based on one-way ANOVA followed by Duncan’s new multiple range test). (**d**) Growth phenotype of 4-week-old plants from the T2 generation of *pGC1*::*BON1/bon1* transgenic line grown at 22°C, 12h/12h L/D. White arrows point to the flat or twisted young leaves of the T2 plants with (left panel) or without (right panel) the *pGC1*::*BON1* transgene respectively (Scale bar = 1 cm).

We examined the apoplastic disease resistance in the chimeric *pGC1*::*BON1/bon1* transgenic lines by dipping and syringe inoculation. Three days after dipping inoculation, the virulent pathogen *Pst* DC3000 amplified to 10^6.0^ CFU mg^-1^ FW in the wild-type Col-0 but reached to only about 10^4.6^ in *bon1-1* (Fig 7A). It amplified to 10^5.2^, 10^5.1^, and 10^5.4^ CFU/mg^-1^ FW in lines ^#^1, ^#^3, ^#^4 in *pGC1*::*BON1/bon1*, respectively (Fig 7A). Growth of *Pst* DC3000 by syringe infiltration was also measured in Col-0, *bon1-1*, and *pGC1*::*BON1/bon1* transgenic plants three days after syringe inoculation at 0 and 3 dpi. The pathogen amplified to about 10^4.4^ CFU cm^-2^ in *bon1-1* while the wild type Col-0 grew almost to 10^6.3^CFU cm^-2^ (Fig 7A). In contrast, *Pst* DC3000 amplified in *pGC1*::*BON1/bon1* by 10^5.5^, 10^5.6^, and 10^5.6^ in lines ^#^1, ^#^3, and ^#^4 respectively, higher than *bon1-1* but lower than Col-0 (Fig 7A). Therefore, the *pGC1*::*BON1/bon1* transgenic plants that differ from *bon1-1* only in guard cell genotype had a reduced apoplastic resistance compared to *bon1-1* (S2 Table).

Expression of defense related genes was then analyzed in *pGC1*::*BON1/bon1* transgenic plants. RNAs were isolated from above-ground plants at 5 weeks old, and qPCR was used to measure gene expression. As expected, the defense marker gene *PR1* was upregulated in leaves of *bon1-1*, at ~55 fold more of the level of wild-type Col-0. Its expression was 18, 25, and 14 fold more of the level of wild type in three *pGC1*::*BON1/bon1* lines, but significantly less than that in *bon1-1* (Fig 7B). Similarly, *SNC1* was more highly expressed in leaves of *pGC1*::*BON1/bon1* transgenic lines compared to the wild type Col-0 but not as high as that in *bon1-1* (Fig 7C). This indicates that the loss of *BON1* function in guard cells contributed significantly to the enhanced apoplastic resistance in the whole plant.

To further define gene expression spatially, we analyzed expression of *PR1* and *SNC1* in mesophyll cells. Both genes were expressed at a higher level in mesophyll cells in all three *pGC1*::*BON1/bon1* lines compared to the wild type, to a similar level in the *bon1-1* plants (Fig 7B and 7C). Therefore a higher expression of *PR1* and *SNC1* in whole leaves of *bon1-1* compared to those of *pGC1*::*BON1/bon1* is likely attributed to mutant *bon1-1* guard cells versus *BON1* wild-type guard cells.

The weaker apoplastic defense of the chimera *pGC1*::*BON1/bon1* plants compared to *bon1-1* was also evident in their milder growth defect which is caused by upregulation of defense responses. Significant differences were observed between *pGC1*::*BON1/bon1* plants and *bon1-1* plants grown under a 12 hr light photoperiod. In the segregating T2 population of two independent *pGC1*::*BON1/bon1* lines (^#^1 and ^#^3), non-transgenic plants (e.g. -/*bon1-1*) all had curved and water-soaked young leaves, whereas plants with the *pGC1*::*BON1* transgene (e.g. *pGC1*::*BON1/bon1*) displayed flatter leaves with a more wild-type appearance (Fig 7D and S4 Fig). This data further supports that the loss of *BON1* function in guard cells (*bon1-1* versus *pGC1*::*BON1/bon1*) significantly enhances plant apoplastic resistance despite of a defective stomatal defense.

### The steady-state stomatal aperture and water potential in the autoimmune mutants

**Fig 8:**
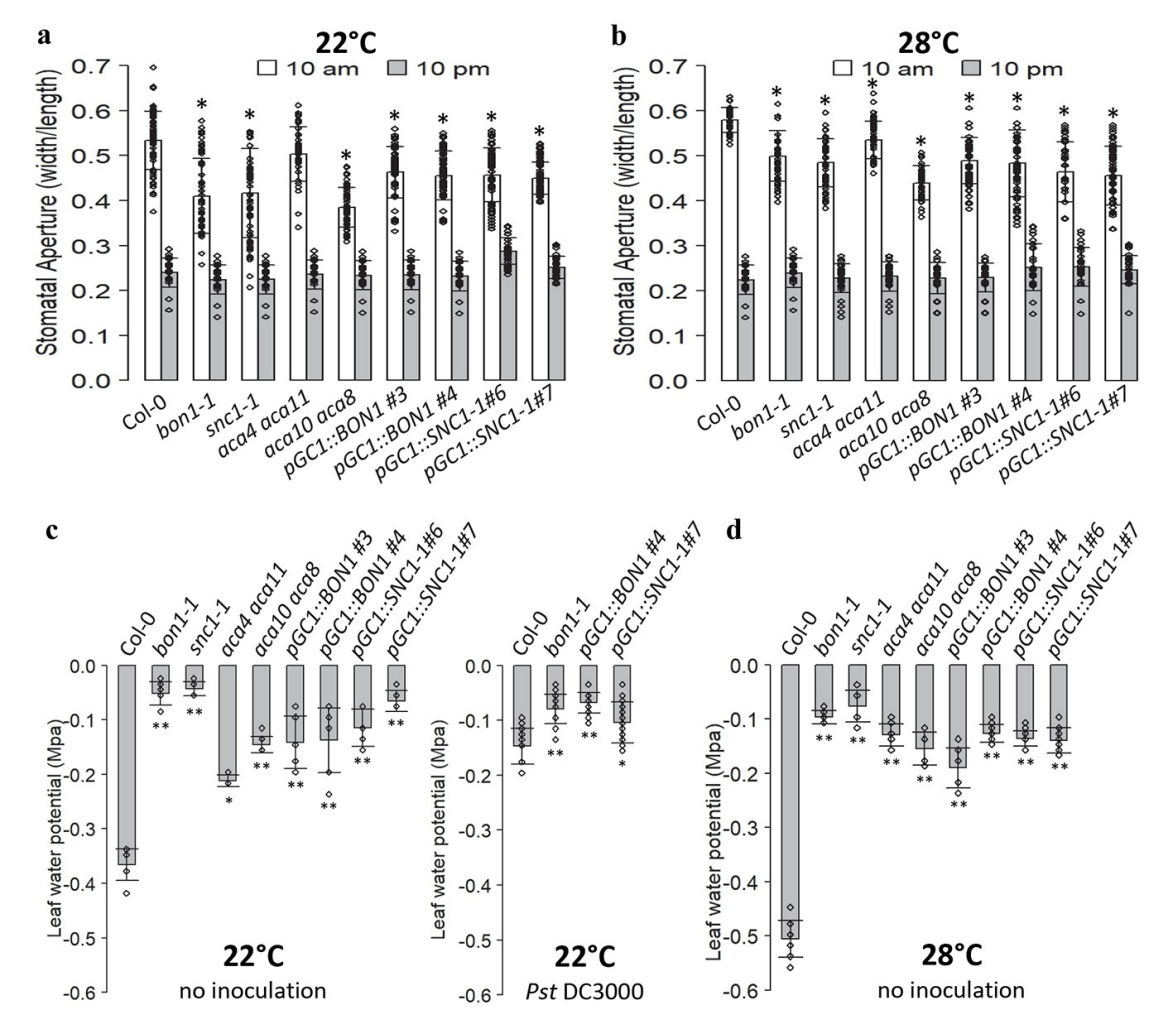
Stomatal aperture and water potential in Col-0, *bon1-1*, *snc1-1*, *aca4 aca11*, *aca10 aca8*, *pGC1*::*BON1/bon1* and *pGC1*::*snc1-1* chimeras. (**a**, **b**) Stomatal apertures measured at 10 am and 10 pm in in wild type, *bon1-1*, *snc1-1*, *aca4 aca11*, *aca10 aca8*, *pGC1*::*BON1/bon1* and *pGC1*::*SNC1-1* plants at 22°C (**a**) and 28°C (**b**). Biological duplicates were averaged, error bars indicate SDs (n = 60 stomata). Asterisks indicate statistically significant differences in stomatal aperture between Col-0 and the mutants at 10 am (*p*< 0.001; based on one-way ANOVA followed by Duncan’s new multiple range test). (**c**) Leaf water potential measured in the indicated plant lines at 22°C before and after 4 hours *Pst* DC3000 spray (OD600 = 0.05). (**d**) Leaf water potential in indicated plant lines at 28°C. For **c** and **d**, plants were grown for 4 weeks and watered well the day before measurements. Asterisks indicate statistically significant differences in water potential between Col-0 and the mutants (*, *p*< 0.05; **, *p*< 0.01; based on one-way ANOVA followed by Duncan’s new multiple range test).

The chimera studies of *BON1* and *SNC1* indicate that *NLR* related activities in guard cells inhibit stomatal defense but at the same time slightly enhance apoplastic resistance. One possibility for this differential defense response is that the short-term response defect in stomatal closure leads to a long-term whole plant physiological change that has a consequence on disease resistance. To test this, we analyzed stomatal aperture under non-pathogenic conditions in *bon1-1*, *snc1-1*, *aca10 aca8*, *aca4 aca11*, and chimeric guard cell lines. All these lines, except for *pGC1*::*BON1/bon1*, have defective stomatal closure response to ABA and pathogens at 22°C but not 28°C (Figs 2–4 and 6). As stomatal aperture changes diurnally, stomata openness was measured at 10 am (ZT3, 7am/7pm cycle) and 10 pm (ZT15, 7am/7pm cycle) when wild-type plants are expected to have open and closed stomata respectively. At 10 pm, all mutants including chimera plants had a stomatal aperture similar to the wild type at both 22°C and 28°C (Fig 8A and 8B), indicating that they are not defective in dark induced closure. At 10 am and 12 pm, all these mutant plants except for *aca4 aca11* had significantly reduced stomatal apertures compared to the wild type at 22°C, with *aca10/8* having the smallest mean aperture (Fig 8A and S5 Fig). Interestingly, all these mutant plants had a reduced aperture compared to the wild type at 28°C, despite having a wild-type stomatal closure response at this temperature (Fig 8B). These data indicate that the steady-state stomatal aperture is not related to short-term stomatal closure responses.

As stomatal aperture may influence water potential and water potential was reported to be associated with disease resistance [42], we measured leaf water potential in the wild type, *bon1-1*, *snc1-1*, *aca10 aca8*, *aca4 aca11* and chimeric guard cell lines at 10 am (ZT3) when stomatal aperture was measured under light at 22°C and 28°C. Variations were observed in three independent experiments, but overall all these mutants had increased water potential compared to the wild type at 22°C and 28°C (Fig 8C and 8D). Therefore, water potential under non-infection condition was largely correlated with steady-state stomata aperture at 22°C. In addition, we measured leaf water potential after pathogen infection in wild type, *bon1-1*, *pGC1*::*BON1/bon1* and *pGC1*::*SNC1-1*. After infection, the wild type and *pGC1*::*BON1* increased while *bon1-1* and *pGC1*::*SNC1-1* decreased water potential compared to non-infection. Nevertheless, *bon1-1*, *pGC1*::*BON1/bon1* and *pGC1*::*SNC1-1* all had significantly higher water potential than the wild type after infection (Fig 8C). Because a decrease of water potential was reported to be associated with disease resistance [42], the increase of water potential at steady state or infection condition may not explain the enhanced apoplastic resistance in these plants.

### ABA response is inhibited in guard cells by *SNC1* activation

**Fig 9:**
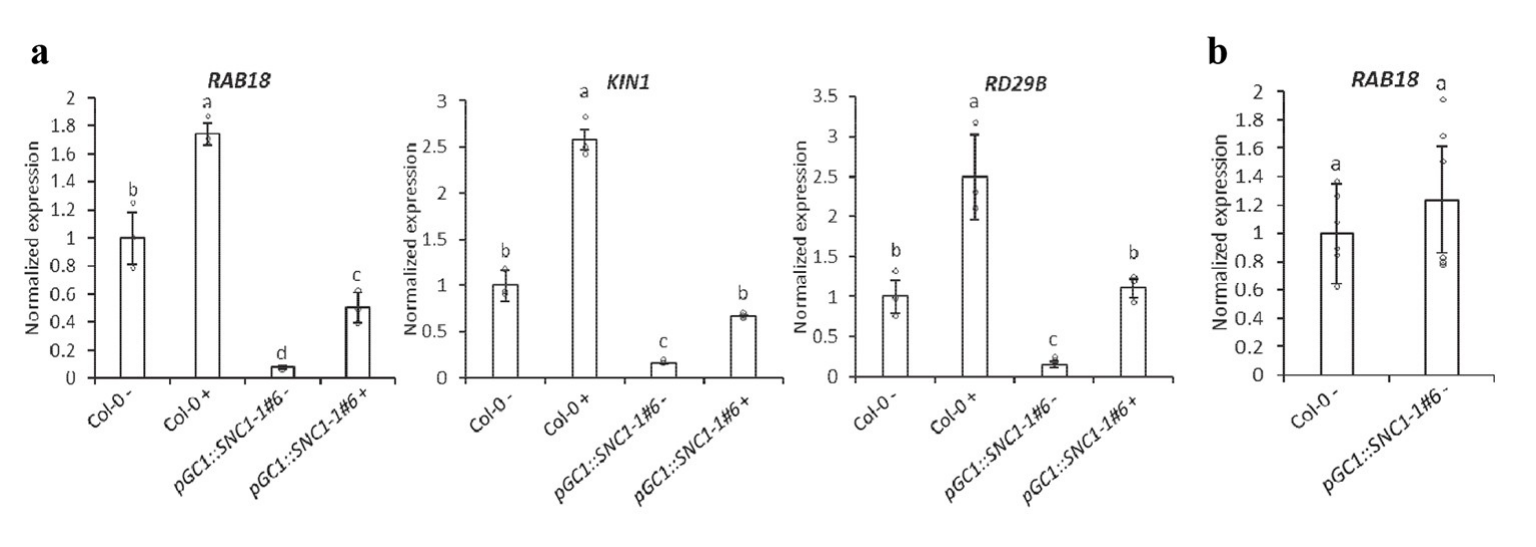
ABA signaling in enriched guard cells assayed by expression of ABA responsive genes. Shown is the transcript abundance of *RAB18*, *KIN1*, and *RD29B* assayed by qPCR in enriched guard cells (**a**) and *RAB18* in whole leaves (**b**) in Col-0 and *pGC1*::*SNC1-1* line with (+) or without (-) *Pst* DC3000 infection. Total RNAs were isolated from purified guard cells and whole rosette leaves of 5-week-old plants. Results were from one of the two independent experiments. The expression was normalized to the expression of a reference gene *ACTIN* in the guard cells of Col-0 without *Pst* DC3000 infection. Values are arithmetic means ± S.E., different letters indicate statistically significant differences between indicated plant lines (p< 0.05, based on one-way ANOVA followed by Student’s t test).

As water potential does not explain stomatal defect, we turned to the possibility of NLR impact on ABA or ABA signaling in the guard cells. Because there is no readily available system to monitor ABA content in guard cells, we enriched guard cells from whole leaves by an ice-blender method and assayed the expression of specific ABA-responsive genes *RAB18*, *KIN1* and *RD29B* [43]. Guard cells from this preparation responded to pathogen infection in a similar manner as those from leaf peels, indicating a functional retention through the enrichment (S6A and S6B Fig). In addition, a very low expression of mesophyll cell specific *CAB3* gene and a high expression of epidermal specific *CER6* gene was found in this enriched guard cell preparation (S6C Fig), indicating a good separation of epidermal cells from the mesophyll cells. RNAs were extracted from the enriched guard cells isolated from the wild-type Col-0 plants and the *pGC1*::*SNC1-1* plants (lines ^#^6) incubated with or without *Pst* DC3000. Quantitative RT-PCR revealed that *GC1* was expressed at a higher level in the enriched guard cell preparations than in the whole leaves (S3C Fig). Quantitative RT-PCR analysis also revealed that the expression of *RAB18* in enriched guard cells of the *pGC1*::*SNC1-1* line was lower than that of wild-type Col-0 without pathogen infection. After infection, *RAB18* expression was increased in both the wild-type and the *pGC1*::*SNC1-1* cells but the expression in *pGC1*::*SNC1-1* was still much lower than that in the wild type (Fig 9A). In the meantime, the expression of *RAB18* in whole leaves was only slightly higher but not significant in *pGC1*::*SNC1-1* than in Col-0 without infection (Fig 9B). This indicates that *SNC1* activation in guard cells inhibits ABA synthesis or signaling in guard cells, which could explain the inhibited stomatal closure response.

We subsequently tested potential impact of NLR genes on stomatal closure movement without constitutive activation in autoimmune mutants. Wild-type stomata were exposed to virulent strain *Pst* DC3000 and avirulent strains *Pst* DC3000 with AvrRpm1 or AvrRps4, respectively, and stomatal aperture was monitored (S7A Fig). At 0.5 hr, while *Pst* DC3000 and *Pst* DC3000 AvrRps4 caused stomatal closure, *Pst* DC3000 AvrRpm1 did not. At 1 hr and 2 hr, stomata were closed to a similar extent for all these strains. At 3 hr, all strains had re-opened stomata, and *Pst* DC3000 AvrRpm1 caused more opening of stomata than other pathogen strains. We subsequently used a loss-of-function mutant *rpm1-3* to differentiate the effects of AvrRpm1 and the NLR gene *RPM1*. The *rpm1-3* mutant had a similar stomatal closure response as the wild type to *Pst* DC3000 AvrRpm1 and *Pst* DC3000 (S7B Fig), indicating that AvrRpm1 but not RPM1, is the cause of a weakened stomatal closure defense. This result is consistent with earlier finding that RIN4, target of AvrRpm1, regulates stomatal aperture during pathogen infection [44].

## Discussion

### Cell autonomous effect of NLR activation on stomatal responses

**Fig 10:**
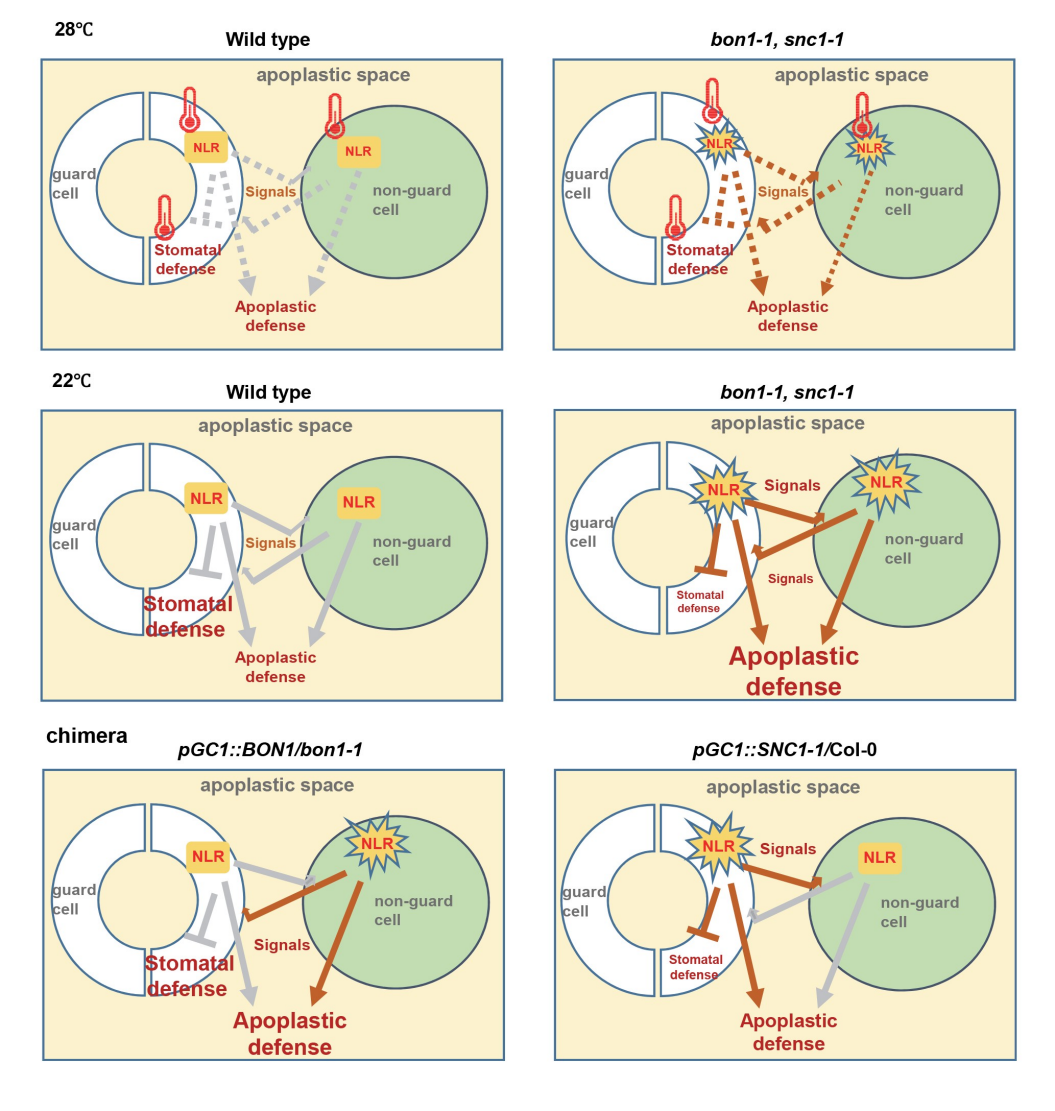
Working model of the connection between stomatal defense and apoplastic defense regulated by NLR genes. NLR activation inhibits stomatal defense in a cell autonomous manner and promotes apoplastic defense in a non-cell autonomous manner. Signals are generated by NLR activation to travel in apoplastic space to influence guard cells and non-guard cells. A higher temperature slows down stomatal defense and inhibits NLR activities independently. Diagrammed are stomatal defense and apoplastic defense in wild type, autoimmune mutants and chimera mutants at 22°C and 28°C. The size of letter indicates the strength/amplitude of the defense. Brown lines indicate strong action, gray lines indicate weak action, and dotted lines indicate no action. Thermometer sign indicates an inhibition from high temperature.

In this study we investigated the effect of NLR activation in guard cells and find that an active form of NLR gene *SNC1*, despite conferring overall enhanced disease resistance, inhibits stomatal closure response to pathogen and compromises stomatal defense. In addition, the effect of NLR activation is cell autonomous for the guard cells. Specific expression of the active form of NLR gene *SNC1* only in guard cells (*pGC1*::*SNC1-1*) is sufficient to cause defective stomatal closure response to pathogens. In support of this, a reverse version of the above mutant, that is the wild type guard cell in the autoimmune *bon1* mutant background (*pGC1*::*BON1/bon1*), exhibits a wild-type stomatal response to pathogen and ABA. Therefore, the stomatal closure response inhibited by NLR activation is cell autonomous for guard cells (Fig 10).

This negative regulation from NLR likely comes from its inhibition of ABA response. Early study reports an inhibitory effect of NLR function on stomatal closure response to ABA [20] and a decreased ABA level by constitutive activation of *SNC1* in whole plant [17]. This study finds that ABA response is inhibited even when *SNC1* is activated only in guard cells (Fig 9). This low ABA response capacity in the guard cells could result in the short-term stomatal closure defect. It is not yet known whether or not all NLR genes could be expressed and activated in guard cells during pathogen infection. Because *SNC1* is expressed in guard cells, some other NLR genes might also be expressed there and be activated by effectors from pathogens. It would be interesting to investigate to what extent their activation in guard cells might affect stomatal defense responses. In addition, NLR activation is usually induced in response to pathogen invasion and constitutive activation of NLR genes may cause long-term effects (such as altered hormone balance) that might be different from the natural plant-pathogen interaction. An inducible system could be helpful to distinguish short-term effects from long-term effects of NLR activation.

### Influence of guard cells on whole plant resistance

One interesting finding of this study is a potential non-cell autonomous effect of guard cell function on whole plant immunity. Expressing the active *SNC1-1* mutant gene only in guard cells induces an enhancement of disease resistance in whole plant. This does not work via the currently defined stomatal defense, because the closure of stomata in response to bacterial pathogens was deficient in NLR activated guard cells, which by itself is expected to lead to a higher susceptibility in the chimera plants. Rather, this suggests a non-cell-autonomous effect of guard cells. We cannot exclude the possibility that active mutant gene *SNC1-1* is expressed at a very low level in non-guard cells due to potential enhancer sequences close to the transgene and therefore the effect is still cell autonomous from non-guard cells. Nevertheless, the more severe growth defect of *bon1-1* compared to *pGC1*::*BON1/bon1* (which is *bon1-1* except for the wild-type guard cells) also supports an enhancement of whole plant resistance from NLR activation in guard cells. The enhanced whole plant resistance induced from guard cell specific activation of NLR comes likely from increased apoplastic resistance, which is supported by the elevated expression of *PR1* gene in mesophyll cells in *pGC1*:*SNC1-1* compared to the wild type. This is also supported by a higher *PR1* expression in mesophyll cells of the *bon1* mutant compared to those of the *pGC1*::*BON1/bon1* plants suggesting an influence from the mutant *bon1* guard cells on mesophyll cells. However, since *BON1* has additional functions other than repressing *SNC1*, this effect of *bon1* guard cells may not be restricted to NLR activation [21,45].

One model for the likely non-cell autonomous effect of guard cell function is that an altered stomatal behavior and consequently altered whole plant physiology are responsible for the enhanced resistance coming from the guard cell defects. The *snc1-1*, *bon1-1*, *pGC1*::*BON1/bon1* and *pGC1*::*SNC1-1* plants have smaller stomatal apertures under light which was likely responsible for the increased water potential at the steady state in these mutants. These mutant plants also had increased water potential after infection. Because a decrease of water potential is shown to enhance disease resistance [42], the increase of water potential under non-infection and infection conditions cannot account for the enhanced disease resistance. The other model for the non-cell autonomous function is that NLR activation in guard cells produce molecules and signals that influence apoplastic resistance. Although guard cells do not form plasmodesmata with other epidermal cells, they may produce and secrete anti-pathogen molecules to apoplasts to enhance resistance (Fig 10). Signaling molecules are also likely generated from guard cells to influence non-guard cells because wild-type mesophyll cells in the *pGC1*::*SNC1-1* transgenic plants have a higher expression of *PR1* than mesophyll cells in the wild type. This signal could be the same signal as in the systemic acquired resistance such as ROS, Ca^2+^ waves, electric signals, or hydraulic waves.

These two models are not mutually exclusive. The opening or closing of stomata could affect the micro-environment within the leaf and alter ROS, NO or ABA levels [46,47]. For instance, high humidity alters stomatal aperture and inhibits systemic responses to pathogens [48]. Our study shows that guard cells, although not capable of transmitting signals through plasmodesmata, might serve as an initiation site of systemic acquired resistance through apoplastic signaling. This hypothesis is supported by the observation that guard cells are often the first site of contact of pathogens in plants.

### Influence on guard cell behavior from NLR activation from non-guard cells

A dis-connection was found between short term stomatal closure response and steady state stomatal aperture. Almost all the mutants we analyzed in this study including those with NLR being activated only in guard cells had a smaller stomatal aperture during the day irrespective of their short-term stomatal closure responses. The cause of smaller aperture under light is not yet known, because a lower ABA or ABA response observed is expected to confer more open stomata. It is likely that the steady state of stomatal aperture might be influenced by molecules generated in apoplastic defense either locally in guard cells or traveled from non-guard cells (Fig 10). For instance, ROS and SA could be produced by guard cells or by non-guard cells and travel to reduce the openness of stomata [47]. Further study should reveal the nature of these signals and whether or not signals from non-guard cells and guard cells are the same.

### Temperature on stomatal immunity

Temperature has a large impact on the interaction between plants and pathogens. High temperature often inhibits ETI resistance mediated by NLR proteins. Prior study observed that elevated temperature upregulates gene expression in PTI [24]. Here we show that high temperature delays but not abolish the stomatal closure response to pathogens (Fig 10). Therefore, high temperature could reduce or slow down stomatal defense in PTI. This reduction of response appears to be immunity specific, because stomatal closure response to ABA is not slowed down by high temperature. This reduction is also not due to a reduced SA response, because SA application induces stomatal closure at a slightly faster rate at high temperature than at normal temperature. This is apparently in contrast to the enhanced defense gene expression during PTI at high temperature. It is therefore possible that temperature inhibits a component downstream of the bifurcation of gene expression and stomatal closure response in PTI. Under high temperature, defects in both apoplastic defense (constitutive active) and stomatal defense (no closure response) are suppressed in these autoimmune mutants. As high temperature inhibits NLR activities, the suppression of the stomatal defense defect likely results from the suppression of NLR activities that induces stomatal closure defect.

In summary, we have uncovered an unexpected guard cell autonomous effect of NLR activation in inhibiting stomatal defense and a non-cell autonomous effect of NLR activation in guard cells or non-guard cells on apoplastic defense. This phenomenon suggests additional layers of modulation of defense responses by NLR genes as well as by cell specific information. Future study should reveal further the cell-specific response and interaction among plant cells during plant pathogen interaction.

## Supporting information

S1 Fig Autoimmune mutants exhibit temperature-dependent disease resistance against *Pst* DC3000. Shown is the growth of *Pst* DC3000 at 0 and 3 dpi in Col-0, *bon1-1*, *snc1-1*, *aca4 aca11* and *aca10 aca8* grown at 22°C (**a, c**) and 28°C (**b, d**) *via* dipping inoculation as log value of cfu per milligram tissue. Values represent three biological repeats, error bars indicate SDs (n = 3). Asterisks indicate statistically significant differences between Col-0 and the mutants (*, *p*<0.05; **, *p*<0.001; student’s t test).
(TIF)

S2 Fig Expression of *SNC1-1* in guard cells does not cause growth defect in Col-0. Shown are 4-week-old plants of Col-0 and the T2 generation of *pGC1*::*SNC1-1* grown at 22°C, constant white light. White asterisks indicate plants without *pGC1*::*SNC1-1*transgene (Scale bar = 2 cm).
(TIF)

S3 Fig Expression of *CAB3, CER6, BON1* and *GC1* in mesophyll cells and the whole leaf in chimera lines. (**a**) The transcript abundance of *CAB3* and *CER6* in mesophyll cell of indicated plant lines assayed by qPCR. (**b**) The transcript abundance of *GC1*, *BON1*, *CAB3* and *CER6* assayed by qPCR in mesophyll cells and whole leaves. Total RNAs were isolated from mesophyll cells and whole rosette leaves of 5-week-old plants. The expression was normalized to the expression of a reference gene *ACTIN2* and relative to their expression in Col-0. Values are arithmetic means ± S.E., different letters indicate statistically significant differences between indicated plant lines (*p*<0.05, based on one-way ANOVA followed by Student’s t test).
(TIF)

S4 Fig Expression of *BON1* in guard cells reduces autoimmunity in *bon1-1*. Shown are 4-week-old plants of Col-0 and the T2 generation of two *pGC1*::*BON1/bon1* transgenic lines grown at 22°C, 12h/12h L/D. White arrows point to the flat young leaves of plants with *pGC1*::*BON1* transgene in their guard cells compared to the twisted young leaves of -/*bon1-1* plants without *pGC1*::*BON1* transgene (Scale bar = 1 cm).
(TIF)

S5 Fig Stomatal aperture in Col-0 and chimeras at 10 am and 12 pm. Shown are stomatal apertures measured at 10 am and 12 pm in the indicated plant lines at 22°C. Results are from one replica representing three biological repeats, error bars indicate SDs (n = 30 stomata). Statistical analysis was performed with one-way ANOVA followed by Tukey-Kramer test (*p*< 0.001).
(TIF)

S6 Fig Epidermal preparation. (**a**, **b**) Stomatal status in epidermal preparations before (a) and after (b) infection by Pst DC 3000. (**c**) The transcript abundance of *GC1*, *CAB3* and *CER6* assayed by qPCR in enriched guard cells (G) and whole leaves (L) with (+) or without (-) *Pst* DC 3000 infection. Total RNAs were isolated from enriched guard cell preps and whole rosette leaves of 5-week-old plants. The expression was normalized to the expression of a reference gene *ACTIN2* and relative to their expression in Col-0 Values are arithmetic means ± S.E., different letters indicate statistically significant differences between indicated plant lines (*p*<0.05, based on one-way ANOVA followed by Student’s t test).
(TIF)

S7 Fig Stomatal responses to avirulent strains of *Pst* DC3000. Stomatal apertures in response to buffer alone (mock) and *Pst* DC3000 strains (with or without indicated effectors) in the Col-0 (**a, left panel of b**) and *rpm1* (**right panel of b**). Results are from one set of experiment, error bars indicate SDs (n = 30 stomata). Asterisks indicate statistically significant differences in stomata aperture between *Pst* DC3000 and avirulent *Pst* DC3000 treatment (*, *p*< 0.001, student’s t test).
(TIF)

S1 Table List of oligos used in this study. (TIF)

S2 Table Summary of stomatal and apoplastic defense phenotypes of mutants in this study. (TIF)
