# Supplementary figures and images for: Cell autonomous and non-autonomous functions of plant intracellular immune receptors in stomatal defense and apoplastic defense
Source: PLoS Pathog. 2019 Oct 25;15(10):e1008094. doi: 10.1371/journal.ppat.1008094 (PMC6834285; doi:10.1371/journal.ppat.1008094)

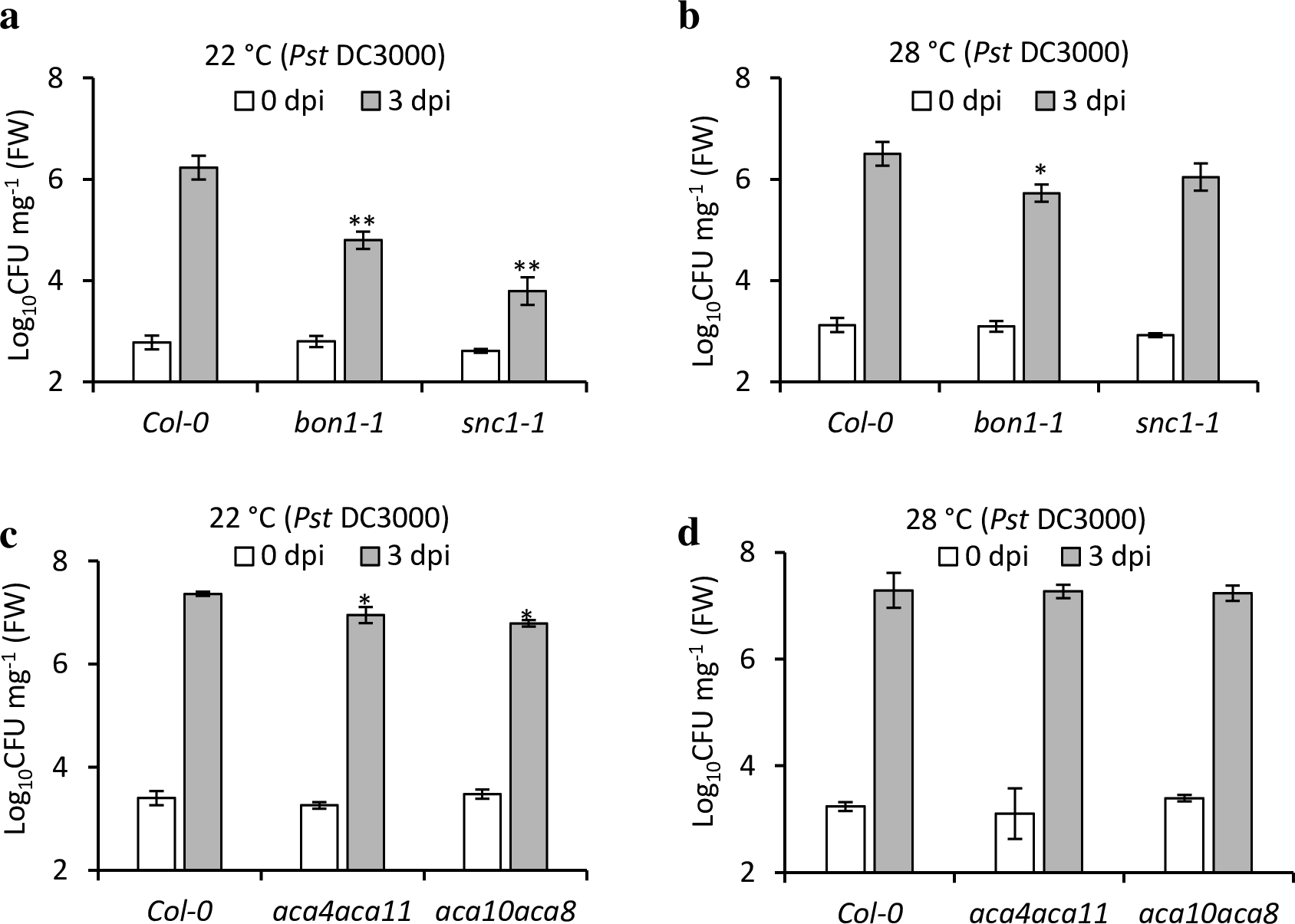

Supplement: S1 Fig — Shown is the growth of Pst DC3000 at 0 and 3 dpi in Col-0, bon1-1, snc1-1, aca4 aca11 and aca10 aca8 grown at 22°C (a, c) and 28°C (b, d) via dipping inoculation as log value of cfu per milligram tissue. Values represent three biological repeats, error bars indicate SDs (n = 3). Asterisks indicate statistically significant differences between Col-0 and the mutants (*, p<0.05; **, p<0.001; student’s t test). (TIF) [file ppat.1008094.s001.tif]

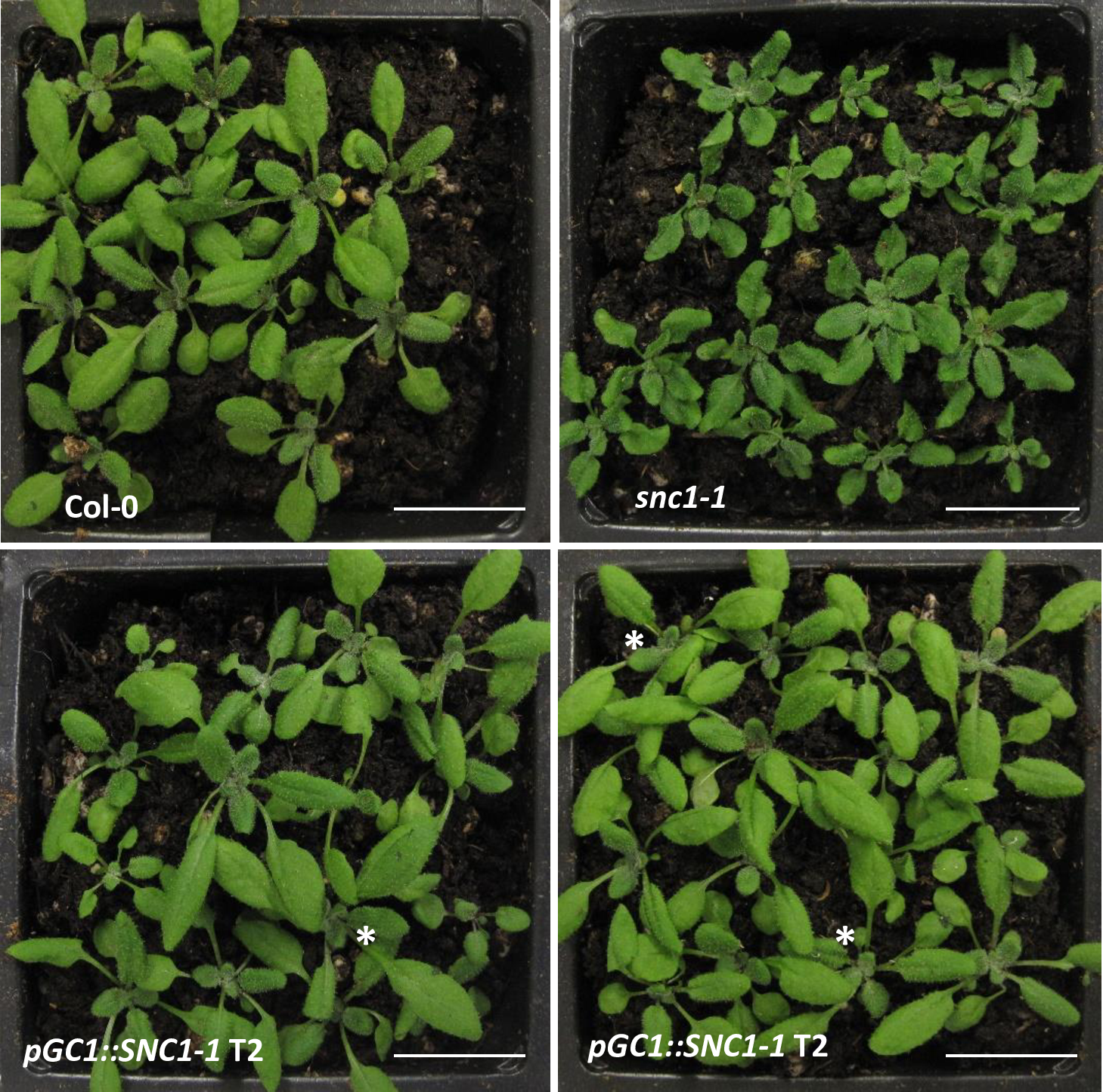

Supplement: S2 Fig — Shown are 4-week-old plants of Col-0 and the T2 generation of pGC1::SNC1-1 grown at 22°C, constant white light. White asterisks indicate plants without pGC1::SNC1-1transgene (Scale bar = 2 cm). (TIF) [file ppat.1008094.s002.tif]

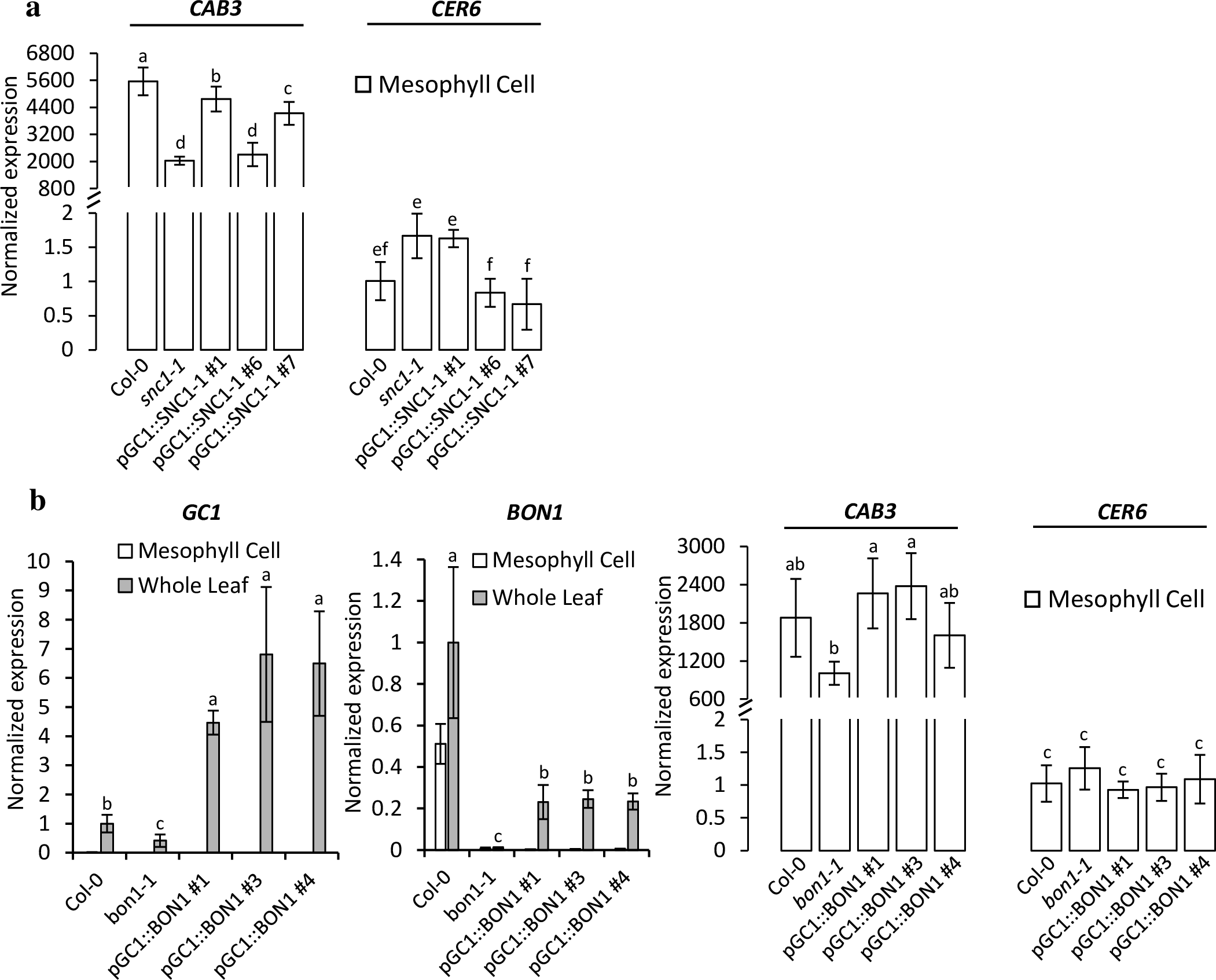

Supplement: S3 Fig — (a) The transcript abundance of CAB3 and CER6 in mesophyll cell of indicated plant lines assayed by qPCR. (b) The transcript abundance of GC1, BON1, CAB3 and CER6 assayed by qPCR in mesophyll cells and whole leaves. Total RNAs were isolated from mesophyll cells and whole rosette leaves of 5-week-old plants. The expression was normalized to the expression of a reference gene ACTIN2 and relative to their expression in Col-0. Values are arithmetic means ± S.E., different letters indicate statistically significant differences between indicated plant lines (p<0.05, based on one-way ANOVA followed by Student’s t test). (TIF) [file ppat.1008094.s003.tif]

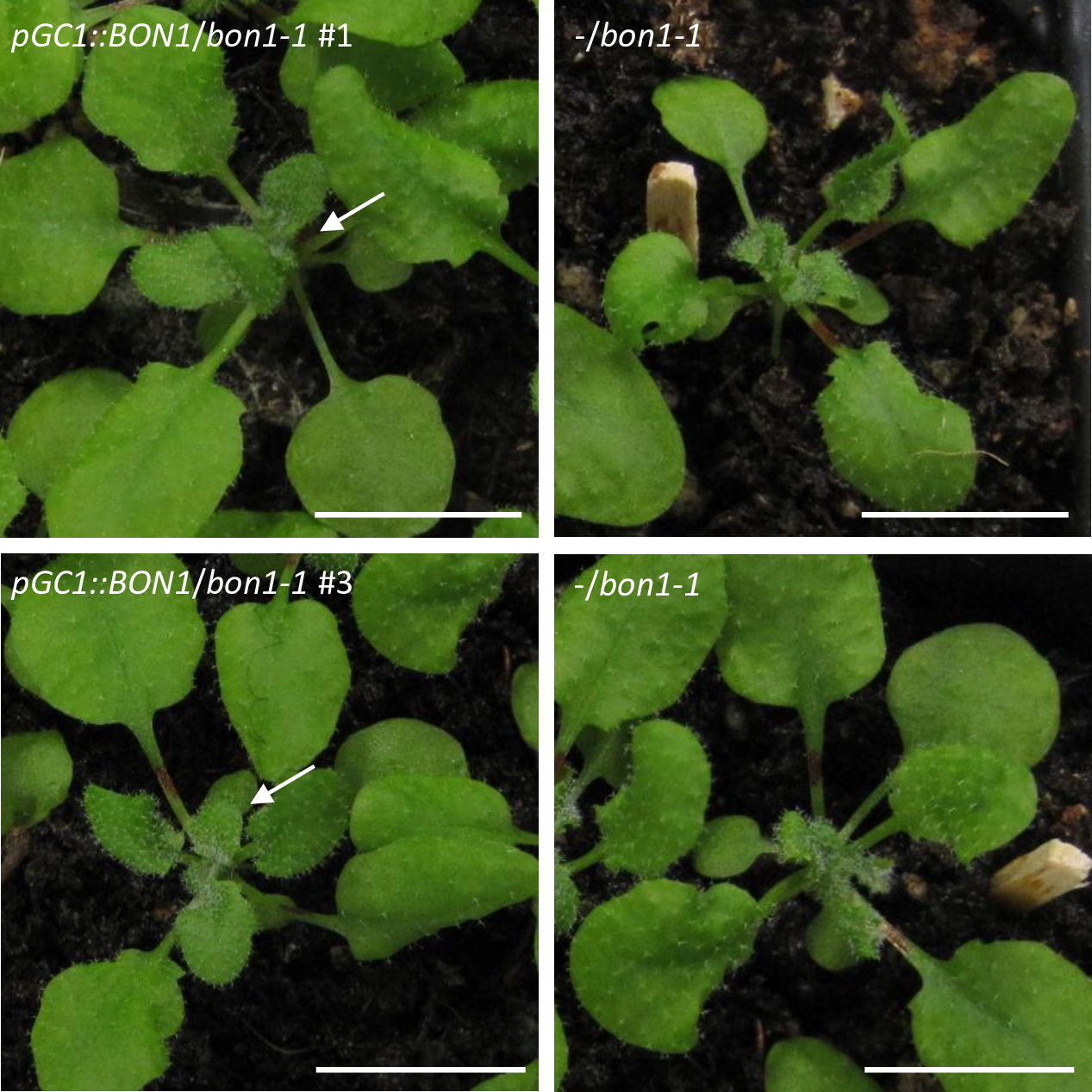

Supplement: S4 Fig — Shown are 4-week-old plants of Col-0 and the T2 generation of two pGC1::BON1/bon1 transgenic lines grown at 22°C, 12h/12h L/D. White arrows point to the flat young leaves of plants with pGC1::BON1 transgene in their guard cells compared to the twisted young leaves of -/bon1-1 plants without pGC1::BON1 transgene (Scale bar = 1 cm). (TIF) [file ppat.1008094.s004.tif]

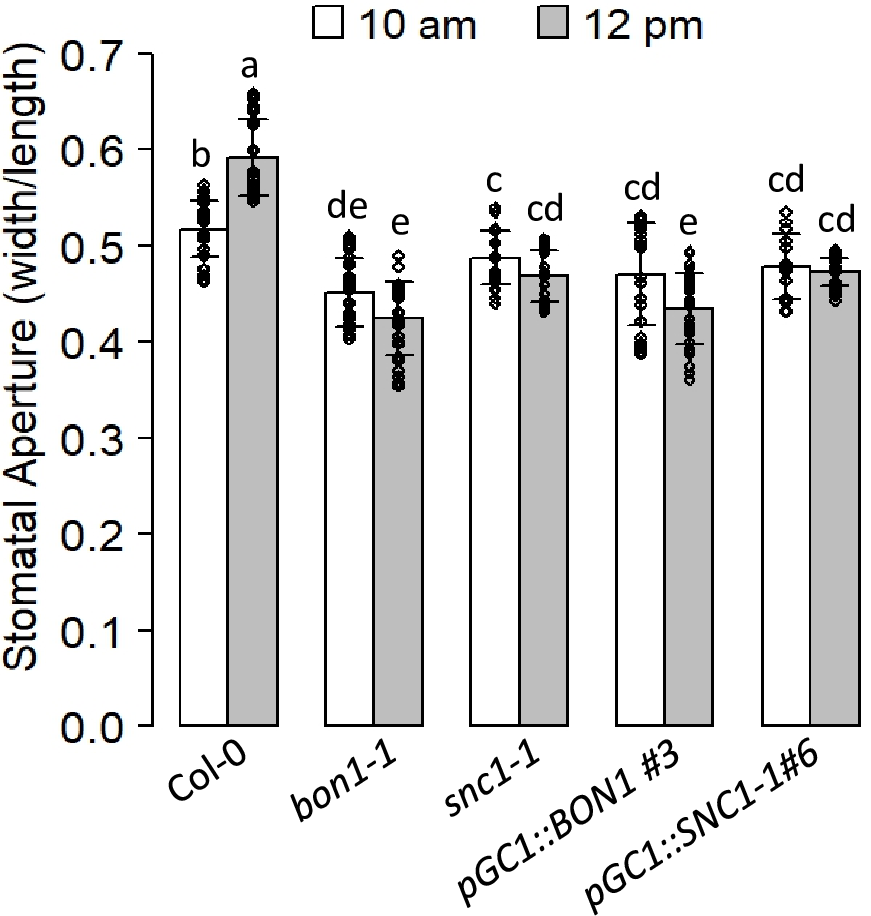

Supplement: S5 Fig — Shown are stomatal apertures measured at 10 am and 12 pm in the indicated plant lines at 22°C. Results are from one replica representing three biological repeats, error bars indicate SDs (n = 30 stomata). Statistical analysis was performed with one-way ANOVA followed by Tukey-Kramer test (p< 0.001). (TIF) [file ppat.1008094.s005.tif]

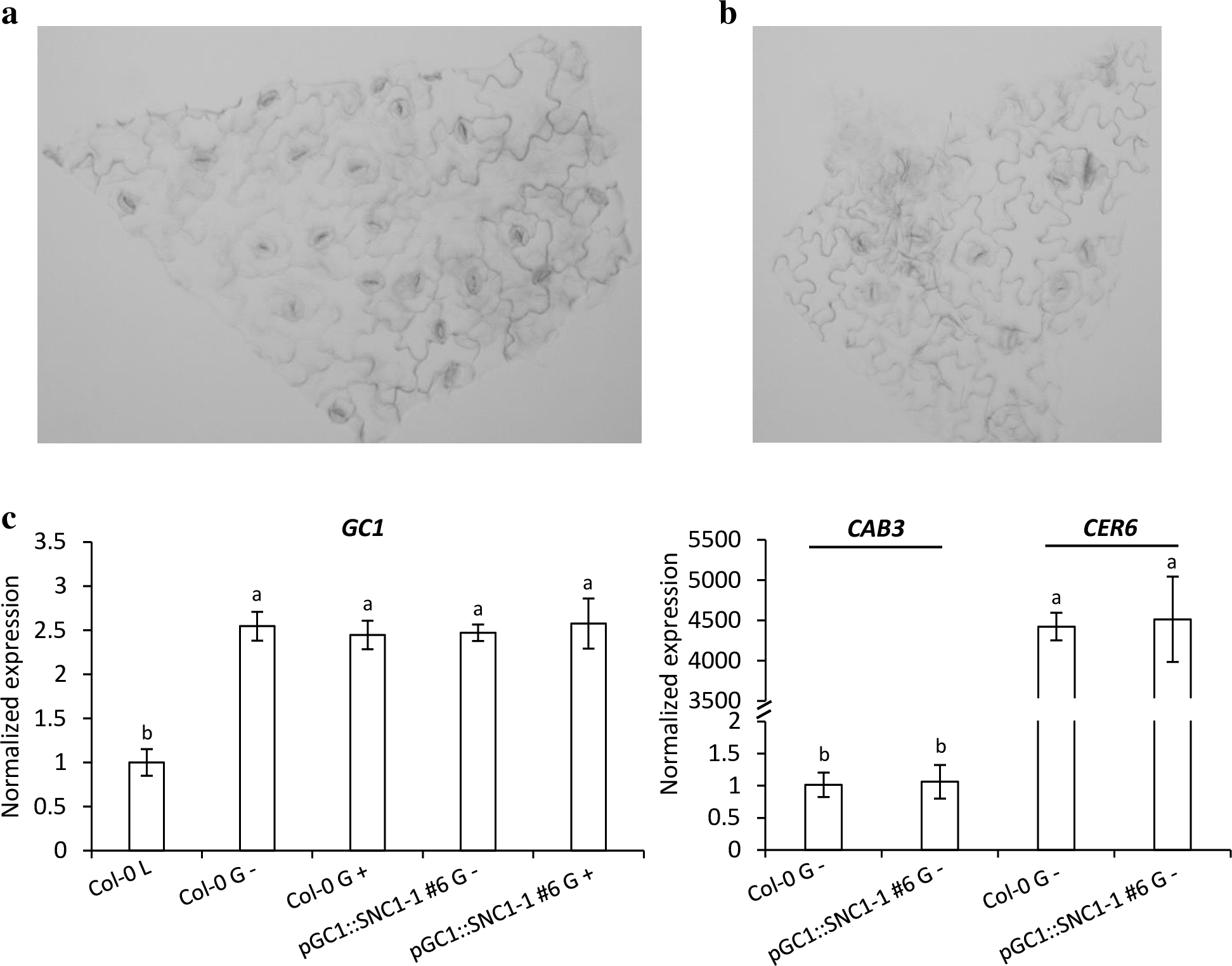

Supplement: S6 Fig — (a, b) Stomatal status in epidermal preparations before (a) and after (b) infection by Pst DC 3000. (c) The transcript abundance of GC1, CAB3 and CER6 assayed by qPCR in enriched guard cells (G) and whole leaves (L) with (+) or without (-) Pst DC 3000 infection. Total RNAs were isolated from enriched guard cell preps and whole rosette leaves of 5-week-old plants. The expression was normalized to the expression of a reference gene ACTIN2 and relative to their expression in Col-0 Values are arithmetic means ± S.E., different letters indicate statistically significant differences between indicated plant lines (p<0.05, based on one-way ANOVA followed by Student’s t test). (TIF) [file ppat.1008094.s006.tif]

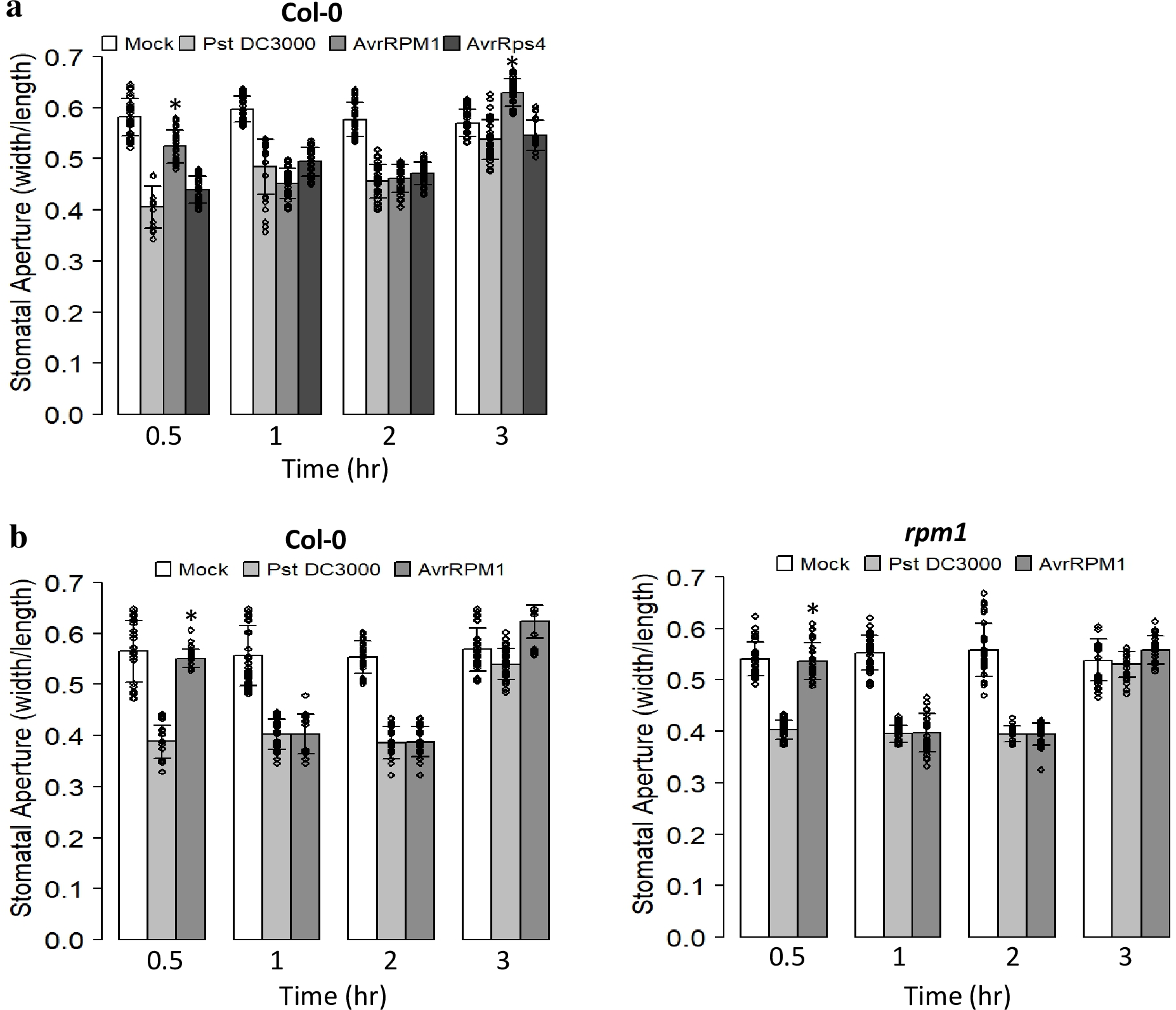

Supplement: S7 Fig — Stomatal apertures in response to buffer alone (mock) and Pst DC3000 strains (with or without indicated effectors) in the Col-0 (a, left panel of b) and rpm1 (right panel of b). Results are from one set of experiment, error bars indicate SDs (n = 30 stomata). Asterisks indicate statistically significant differences in stomata aperture between Pst DC3000 and avirulent Pst DC3000 treatment (*, p< 0.001, student’s t test). (TIF) [file ppat.1008094.s007.tif]

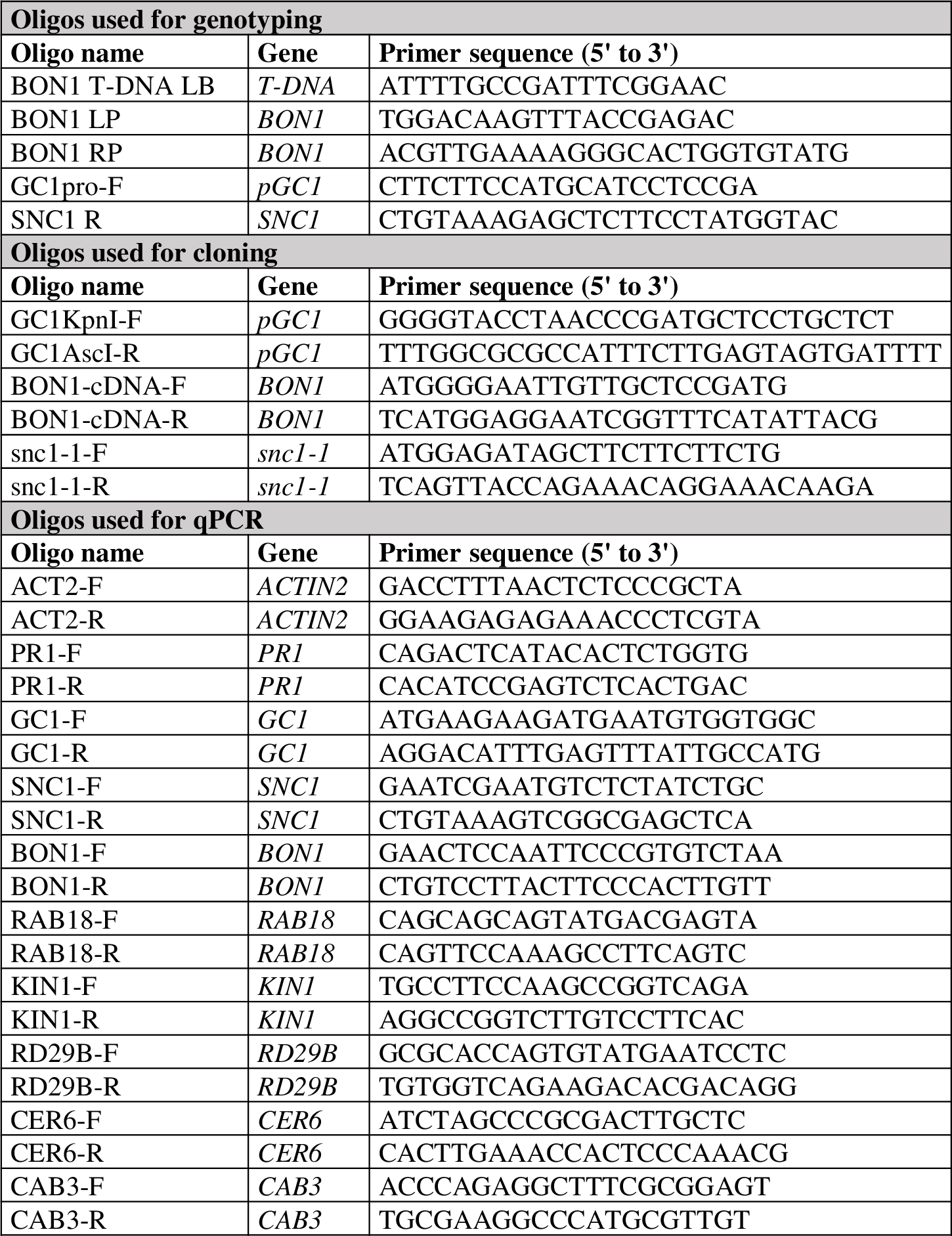

Supplement: S1 Table — (TIF) [file ppat.1008094.s008.tif]

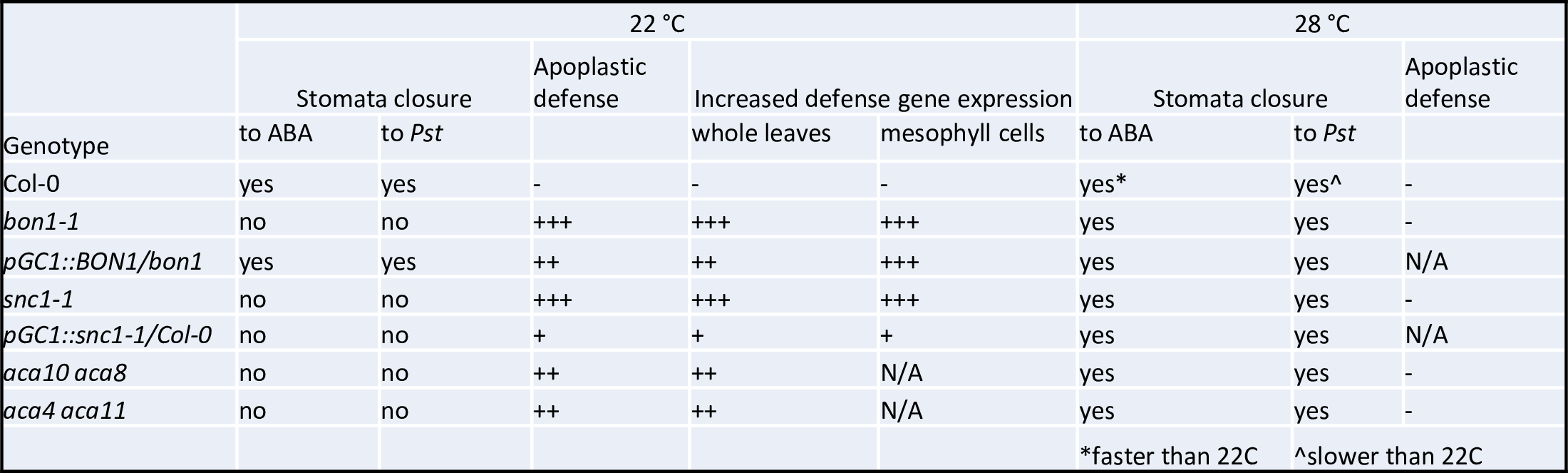

Supplement: S2 Table — (TIF) [file ppat.1008094.s009.tif]
